# Iridium Complexes of a Triazole‐Derived Pincer Ligand: Synthesis, Reactivity, and Transfer Dehydrogenation Catalysis

**DOI:** 10.1002/chem.70909

**Published:** 2026-04-01

**Authors:** Jesvita Cardozo, Ouchan He, Aaron Prenzlow, Xinyang Peng, Kallol Ray, Thomas Braun

**Affiliations:** ^1^ Department of Chemistry Humboldt‐Universität zu Berlin Berlin Germany

## Abstract

An asymmetric *^t^* ^Bu^POCN_Triaz_ type pincer ligand containing a triazole arm was developed and used to generate iridium pincer complexes with different oxygen atom binding ancillary ligands. The structure and reactivity of the complexes [(*^t^* ^Bu^POCN_Triaz_)Ir(H)(O_2_CCH_3_)], [(*^t^* ^Bu^POCN_Triaz_)Ir(H)(acac)], and [(*^t^* ^Bu^POC)Ir(H)(O_2_CCF_3_)] and its cationic derivatives were studied. The complexes could be successfully employed in the catalytic transfer dehydrogenation of alkanes as well as of unsaturated heterocycles.

## Graphical abstract

Ir pincer complexes bearing a triazole architecture in the pincer backbone and O‐ancillary chelating ligands are presented. These were employed in the catalytic transfer dehydrogenation of alkanes as well as of saturated heterocycles. Reactivity of these complexes with silver salts and Lewis acids produced various cationic iridium complexes.

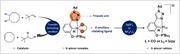

## 1 Introduction

Alkenes serve as versatile raw materials for industry; hence both the functionalization of alkenes as well as generation of alkenes via alkane dehydrogenation has been extensively explored, especially using pincer chemistry [1, 2, 3]. For the catalytic dehydrogenation of alkanes to alkenes, iridium pincer complexes with several PCP, POCOP, and NCP type pincer ligands have been studied [3, 4, 5, 6, 7, 8]. While the PCP and its isoelectronic system POCOP are found to be the more effective catalysts [9, 10, 11, 12], several hurdles like subsequent isomerization of the obtained alkenes or product inhibition have limited applications of these systems. In literature, several alternative pincer systems have thus been explored including unsymmetrical NCP and POCN pincer ligand complexes [13, 14, 15, 16, 17, 18], where the N‐donor is usually a sp^2^ imine N‐atom of imines [16] or of heterocycles like pyridine [14], oxazole [15], benzoquinone [13], and pyrazole [17]. Compared to its phosphorous counterpart, the hard N‐donor shows interesting properties like hemilability and can possibly confer ligand cooperativity [17, 19, 20]. While the triazole ring is known to be easily accessible using the click reaction [21, 22, 23], it has never been applied as ligand building block in iridium‐pincer complexes. Modification of triazole containing pincer ligands can be easily envisioned via CuAAC (copper catalyzed azide‐alkyne cycloaddition) [24], which can be employed to tune the electronic properties of the N‐donor in an iridium complex.

While most of these iridium‐pincer complexes have ancillary chlorido ligands, very few are reported with oxygen atoms as binding ligands, for example, acetate [16, 25, 26]. The chlorido containing iridium complexes often need a hydrogen atmosphere at high temperatures for metalation of the pincer ligand precursors [27, 28, 29, 30]. On the other hand, formation of the corresponding acetato complexes requires only an appropriate iridium metal precursor under inert conditions. In contrast to a chlorido ligand, the acetato ligand offers chelation at the iridium center, which could in principle be beneficial for any catalytic reaction cycle [25, 26, 31]. While the acetato ligands often chelate to a metal center, trifluoracetato ligand chelation is weak. Like their chlorido counterparts, these complexes are often coordinatively unsaturated and are more reactive along with a higher sensitivity to air and moisture [5]. However, acetylacetonate ligands [32, 33] show bidentate coordination at the metal center, providing a coordinatively saturated iridium centers and often more stable complexes.

Another interesting concept in the realm of iridium pincer complexes for catalytic dehydrogenations, is obtaining cationic iridium pincer catalysts by removal of one of the ancillary ligands [34, 35, 36, 37, 38]. Literature reports for the generation of such cationic complexes employ iridium dihydrido or iridium hydrochloride complexes as starting materials [39, 40]. For complexes with oxygen containing ancillary ligands, differences in reactivities are expected. However, to the best of our knowledge, no such literature reports are found.

Herein we report on 1,2,3‐triazole‐ based iridium‐pincer complexes bearing different oxygen containing chelating ligands. The synthesized compounds [(*^t^* ^Bu^POCN_Triaz_)Ir(H)(O_2_CCH_3_)] {OAc = acetato} (**1**) and [(*^t^* ^Bu^POCN_Triaz_)Ir(H)(acac)] {acac = acetylacetonato} (**3**) were employed in the catalytic transfer dehydrogenations of alkanes and unsaturated heterocyles. When the complexes were transformed into cationic compounds on using silver salts unique cyclometallation and electron transfer pathways were discovered.

## 2 Results and Discussion

### 2.1 Synthesis of [(*^t^* ^Bu^POCN_Triaz_)Ir(X)(H)] (X = O_2_CCH_3_, O_2_CCF_3_, acac)

**SCHEME 1:**
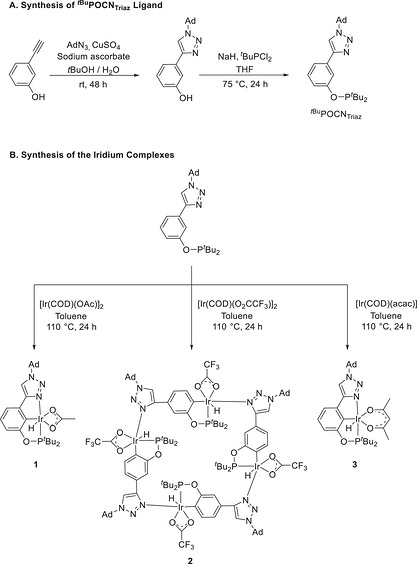
Synthesis of the *^t^* ^Bu^POCN_Triaz_ ligand precursor (A) and metalation with iridium complex precursors (B).

An asymmetric *^t^* ^Bu^POCN_Triaz_ ligand precursor, comprising of a triazole *N*‐donor handle as well as a bulky phosphinite donor was synthesized. The triazole group was prepared via a copper mediated click reaction of 3‐ethynylphenol and 1‐azidoadamantyl. The obtained intermediate was then treated with di‐*tert*‐butylchlorophosphine to obtain the *^t^* ^Bu^POCN_Triaz_ pincer ligand precursor as described in Scheme 1.

Various iridium metal precursors containing anionic oxygen‐donor ligands were employed for complexation. Treatment of the pincer ligand precursor *^t^* ^Bu^POCN_Triaz_ with [Ir(COD)(O_2_CCH_3_)]_2_ produced the complex [(*^t^* ^Bu^POCN_Triaz_)Ir(H)(O_2_CCH_3_)] (**1**) (Scheme 1). The complex shows a doublet for the iridium bound hydrido ligand at −30.50 ppm (*J*_P,H_ = 22.5 Hz) and a signal for the proton of the triazole heterocycle at 7.85 ppm in the ^1^H NMR spectrum. The phosphinite moiety reveals a peak at 157 ppm in the ^31^P{^1^H} NMR spectrum. The ATR IR spectrum shows a band for an Ir─H moiety at 2274 cm^−1^, and absorption bands at 1537 and 1453 cm^−1^ indicating a chelating acetate [16, 41, 42].

Attempts to obtain a fluorinated [(*^t^* ^Bu^POCN_Triaz_)Ir(H)(O_2_CCF_3_)] trifluoroacetato pincer complex failed. Instead, the tetrameric complex [(*^t^* ^Bu^POC)Ir(O_2_CCF_3_)(H)]_4_ (**2**) was generated, wherein cyclometallation at the aromatic ring at the *para* position of triazole moiety occurred. The phosphinite arm and the aromatic ring are bound to the iridium center in a bidentate fashion, with the trifluoroacetate ligand in *trans*‐position to the phosphinite arm. The triazole N‐donor atom of the ligand did not participate in pincer complex formation, but coordinates instead to the next [(*^t^* ^Bu^POC)Ir(H)(O_2_CCF_3_)] unit [43, 44, 45]. This is evident from the splitting pattern in the aromatic region of the ^1^H NMR spectrum, wherein only one *ortho* coupling was observed for the signals at 7.32 ppm (d, *J*_H,H_ = 7.7 Hz) and 6.70 ppm (dd, *J*_H,H_ = 7.7 Hz, 1.4 Hz), and an additional doublet (1.4 Hz) was obtained at 7.20 ppm. The ^1^H NMR spectrum of complex **2** displays the signals for the iridium hydrido ligand at −31.98 ppm (*J*_P,H_ = 28.5 Hz) and for the triazole proton at 7.76 ppm, while the ^31^P{^1^H} NMR spectrum shows a singlet at 153 ppm. A ^1^H NMR NOESY spectrum further reveals a contact of the hydrido ligand with the aromatic proton that shows a signal at 7.20 ppm, which is possible for a tetrameric structure in solution. (See Figure S14) Poor quality crystals of **2** also revealed the tetrameric structure. (See Figure S15) The IR spectrum of the solid compound is comparable to the one in *ortho*‐dichlorobenzene, thereby confirming the tetrameric structure in solution. It exhibits stretching bands for the trifluoroacetate at 1625 and 1453 cm^−1^, indicating chelation [41, 46]. (See Figures S12 and S13)

**SCHEME 2:**
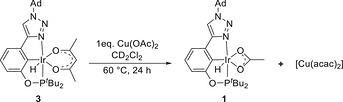
Ligand exchange reaction of **3**.

Treatment of [Ir(COD)(acac)] {acac = acetlyacetonato} with *^t^* ^Bu^POCN_Triaz_ resulted in the formation of [(*^t^* ^Bu^POCN_Triaz_)Ir(H)(acac)] (**3**). Iridium pincer complexes bearing the bidentate acac ligand, have, to the best of our knowledge, previously not been described in literature. The bidentate coordination mode leads to an octahedral complex, which is remarkably stable to air and moisture. The ^1^H NMR spectrum of **3** displays a singlet at 7.74 ppm for the triazole arm of the pincer; the signal for the enolic proton of the acac ligand is observed at 5.23 ppm and a doublet was found for the iridium hydride at −26.06 ppm (*J*_P,H_ = 23.8 Hz). Interestingly, heating complex **3** with copper (II) acetate led to a ligand exchange reaction producing complex **1,** with 80% yield (Scheme 2).

**FIGURE 1:**
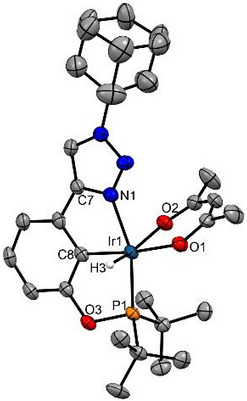
Molecular structure of [(*^t^* ^Bu^POCN_Triaz_)Ir(acac)(H)] (**3**).

The molecular structure of complex **3** was determined by single X‐ray diffraction analysis (Figure 1). The structure revealed an Ir─N bond length of 2.109(4) Å, which is comparable to the Ir─N bond lengths obtained for literature reported (POCN)Ir pincer complexes [13, 14, 15, 47]. The bulky adamantyl group of the triazole ring in the meridional coordinated pincer ligand faces away from the iridium center, while the *t*Bu groups at the phosphinite arm contribute to the bulk around the metal center. The hydride was located in the difference Fourier map at the axial position of the octahedral complex with a Ir‐H bond distance of 1.30(8) Å, while the bidentate acac ligand coordinates with Ir─O bond lengths of 2.192(4) and 2.126(4) Å.

### 2.2 Reactivity of [(*^t^* ^Bu^POCN_Triaz_)Ir(X)(H)] (X = O_2_CCH_3_, O_2_CCF_3_, acac) With CO

**SCHEME 3:**
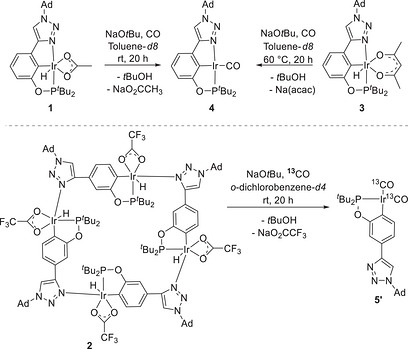
Reactivities of **1**, **2**, and **3** toward CO or ^13^CO in the presence of NaO*t*Bu.

The produced complexes could be transformed into Ir(I) carbonyl complexes by reductive elimination in the presence of NaO*t*Bu and CO. It can be presumed that the hydrido ligand is abstracted in presence of the strong base NaO*t*Bu to induce the reductive elimination step producing *t*BuOH and CH_3_CO_2_Na [18, 26, 47, 48, 49, 50]. Thus, the reaction of complex **1** with CO proceeded at room temperature to give complex [(*^t^* ^Bu^POCN_Triaz_)Ir(CO)] (**4**) (Scheme 3). Complex **3** produced the same species when treated with CO, however heating of the reaction to 60°C was necessary. Complex **4** was identified by a singlet at 197 ppm [37, 50] in the ^13^C{^1^H} NMR spectrum for the carbonyl ligand and a singlet at 184 ppm in the ^31^P{^1^H} NMR spectrum. The ATR IR spectrum showed the CO stretching band at 1934 cm^−1^ [37, 50].

In contrast, a reaction of complex **2** with CO in the presence of a base, led to the formation of the dicarbonyl complex **5**, the structure of which could be elucidated by repetition of the reaction with labelled ^13^CO to yield **5’** (Scheme 3). The ^13^C{^1^H} NMR spectrum of **5’** shows a doublet of doublets at 186 ppm with couplings of *J*_P,C_ = 105 Hz and *J*_C,C_ = 3.8 Hz for the carbonyl ligand in the *trans* position to the phosphinite arm, and a doublet of doublets at 187 ppm with coupling constants of *J*_P,C_ = 7.5 Hz, and *J*_C,C_ = 3.8 Hz for the *cis* carbonyl ligand. The ^31^P{^1^H} NMR spectrum exhibits accordingly a doublet of doublet at 188 ppm. The ATR‐IR spectrum of **5** displays two carbonyl stretching bands at 2037 and 1967 cm ^−1^ [37, 50].

### 2.3 Synthesis of Cationic Complexes

**SCHEME 4:**
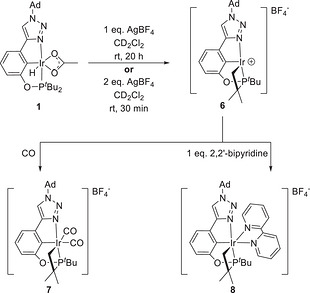
Reactivity of **1** with AgBF_4_.

Attempts were made to produce cationic iridium pincer‐ complexes from **1** and **3**. Treatment of complex **1** with silver tetrafluoroborate produced the cationic complex **6** overnight (Scheme 4). The ^31^P{^1^H} NMR spectrum of **6** shows a peak at 86 ppm, and the ^1^H NMR spectrum reveals signals for inequivalent *tert*‐butyl groups at 1.42 (d, *J*_P,H_ = 16.6 Hz), 1.39 (d, *J*_P,H_ = 18.4 Hz) and 1.02 ppm (d, *J*_P,H_ = 14.4 Hz), indicating cyclometalation of the phosphinite arm at the iridium centre. Note that iridium pincer complexes are known for intramolecular C─H activation, especially for the phosphinite arms containing *t*Bu groups [35, 38, 51, 52, 53]. Although **6** was stable in solution, it decomposed during attempts of filtration or further purification. However, the identity of complex **6** could be further supported by addition of CO or 2,2′‐bipyridine to give species **7** or **8**, respectively. On using ^13^CO the isotopologue **7’** was produced, for which the ^13^C{^1^H} NMR spectrum shows signals at 170.4 ppm (dd, *J*_C,C_ = 2.4 Hz, *J*_C,P_ = 1.0 Hz) and 161.1 ppm (d, *J*_C,C_ = 2.4 Hz). The rather small coupling constants indicate that the carbonyl groups are in a *cis*‐position to the phosphinite arm. The carbonyl absorption bands were found in the IR spectrum at 2069 and 2031 cm^−1^.

Interestingly, the cyclometalated complex **6** could alternatively be obtained in an independent reaction of **1** with two equivalents of AgBF_4_ within 30 min (Scheme 4). Mechanistically, it can be presumed that the first equivalent of AgBF_4_ abstracts the acetato ligand. With the second equivalent of AgBF_4_ we suggest a PCET process as it is outlined further below for the chemistry of **3** [53, 54, 55]. This leads to the generation of HBF_4_ and Ag, where the generation of the latter is indicated by the formation of a grey precipitate. HBF_4_ converts in fluorosilicates, which was observed by NMR spectroscopy (see also below and Supporting Information Figures S1, S2, S3).

**SCHEME 5:**
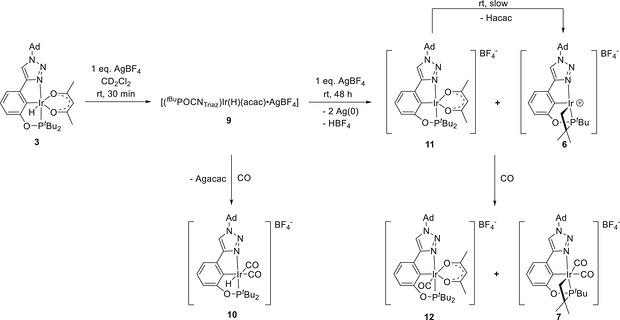
Reactivity of **3** with AgBF_4._

In contrast, treatment of complex **3** with one equivalent of silver tetrafluoroborate produced a species **9**, the structure of which is unknown, but an interaction of the Ag^+^ ion with the acac ligand is suggested. The ^1^H NMR spectrum exhibits a doublet at −29.6 ppm (d, *J*_P,H_ = 22.4 Hz) for the hydrido ligand and the ^31^P{^1^H} NMR spectrum showed a signal at 154 ppm (Scheme 5). Note that the data are very similar to those of the starting complex **3** having signals at −26.0 (d, *J*_P,H_ = 22.4 Hz) and 156.7 ppm. However, a free coordination site in the *trans*‐position to the hydride would lead to a large downfield shift of the iridium‐hydride resonance [38, 56], which is not observed. The ESI‐MS (positive mode, MeCN eluent) of the reaction solution displays *m*/*z* = 673.5 for [(*^t^* ^Bu^POCN_Triaz_)Ir(H) + MeCN]^+^ *and m*/*z* = 714.5 for [(*^t^* ^Bu^POCN_Triaz_)Ir(H) + 2MeCN]^+^.

Although compound **9** was stable in solution, attempts for isolation failed. Nevertheless, if carbon monoxide was added, the formation of the stable cationic dicarbonyl complex [(*^t^* ^Bu^POCN_Triaz_)Ir(H)(CO)_2_][BF_4_] (**10**) was observed. Complex **10** shows a resonance for the hydrido ligand at −10.0 ppm (d, *J*_P,H_ = 16.8 Hz) in the ^1^H NMR spectrum and a signal at 170 ppm in the ^31^P{^1^H} NMR spectrum. The IR spectrum of **10** reveals two CO stretching bands at 2111 and 2077 cm^−1^, while the ^13^C NMR spectrum of its **10’** isotopologue showed two peaks at 167 ppm (ddd, *J*_C,H_ (*cis*) = 5.4 Hz, *J*_C,C_ = 2.4 Hz, *J*_C,P_ = 1.2 Hz) and at 166 ppm (ddd, *J*_C,H_ (*trans*) = 55.9 Hz, *J*_C,C_ = 2.4 Hz, *J*_C,P_ = 3.6 Hz) [38].

To elucidate the observed reactivity further, another equivalent of AgBF_4_ was added to complex **9** (Scheme 5). Interestingly, a new intermediate **11** and the cyclometalated complex **6** were obtained after 48 h. A ^1^H NMR spectrum of the reaction mixture after 5 days, showed no signals in the hydride region, while the ^31^P{^1^H} NMR spectrum showed peaks at 133 and 86 ppm, for **11** and **6**, respectively. Addition of CO led to the formation of the carbonyl complexes **12** and **7**, which show signals in the ^31^P{^1^H} NMR spectra at 150 and 96 ppm, respectively, while masses of 686.21 for [**7‐MeCN**], 699.24 for [**7‐(MeCN)_2_**] and 758.27 for **12** in the ESI‐MS support its identity. (See Figures S4 and S5) The CO absorption bands are observed in the IR spectra at 2059 cm^−1^ for complex **12** and at 2069 and 2031 cm^−1^ for **7** as mentioned above.

The addition of AgBF_4_ to **9** also results in the formation of a grey Ag‐precipitate and fluorosilicates with disappearance of the residual silicon grease signal in the ^1^H NMR spectrum, which indicates generation of HBF_4_ in the reaction. As suggested earlier, and also corroborated by CV studies, a proton‐coupled electron‐transfer (PCET) reaction [35, 53, 57] might occur at **9** to produce a [(*^t^* ^Bu^POCN_Triaz_)Ir(II)]•[BF_4_] intermediate, Ag and HBF_4_, followed by the formation of **11** and Ag by reaction with the Ag(acac) [53]. Complex **11** further undergoes cyclometallation to produce complex **6,** accompanied by elimination of Hacac. The latter reacts with HBF_4_ to give (acetylacetonato)difluoroboron BF_2_acac, which was detected by GCMS analysis of the reaction mixture and showed a signal in the ^11^B{^1^H} NMR spectrum at 0.6 ppm [58, 59]. (See Figures S6–S9)

The cyclic voltammogram of **3** shows a quasi‐reversible oxidation wave in CH_2_Cl_2_, with an oxidation wave (*E*_p,a_) at 0.407 V, and a reduction wave (*E*_p,c_) at 0.301 V; all potentials are referenced vs the Fc^+/o^ couple. Notably, the current corresponding to the reduction wave increases at higher concentrations of externally added acids, thereby, becoming more reversible at lower pH values. (See Figures S16, S17) This is consistent with the fact that the Ir species generated by electrochemical one‐electron oxidation of **3** by cyclovoltammetry decomposes by a deprotonation step, and can only be stabilized at higher proton concentrations. This is also consistent with the mechanism proposed for the conversion of **3** to **11** in presence of two equivalents of AgBF_4_, where an oxidation process of **9** is involved with the concomitant loss of H^+^. The first equivalent of AgBF_4_ is used to form **9**; PCET is only involved in the second step to generate **11** and Ag.

**SCHEME 6:**
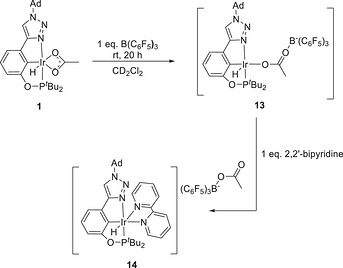
Reactivity of **1** with 1 equivalent of B(C_6_F_5_)_3_.

In order to study the synthesis of cationic complexes further, **1** was treated with B(C_6_F_5_)_3_ (Scheme 6). The bulky Lewis acid led to an interaction with the acetato ligand to yield complex **13** [25, 60]. Notably, no cyclometallation was observed. Compound **13** was characterized by NMR spectroscopy, where the ^1^H NMR spectrum shows a broad upfield resonance at –43 ppm for the hydrido ligand *trans* to a vacant coordination site [38, 56]. A low temperature ^1^H NMR spectrum of **13** at −40°C resolved this broad peak into a distinctive doublet with a coupling constant of *J*_P,H_ = 22 Hz. (See Figure S10) The ^11^B{^1^H} NMR spectrum of **13** also displays a broad peak at −1.6 ppm, possibly suggesting a dynamic interaction of the Ir center with the [B(OAc)(C_6_F_5_)_3_] fragment [60, 61]. When 2,2′‐bipyridine was added to **13,** complex **14** was produced, which reveals a sharp hydride resonance at −20.3 ppm (d, *J*_P,H_ = 24 Hz) in the ^1^H NMR spectrum. The ^11^B{^1^H} NMR spectrum shows a peak at −5.1 ppm, which further confirms the presence of the [B(OAc)(C_6_F_5_)_3_]^−^ ion [61, 62].

Unfortunately, all the coordinatively unsaturated cationic species were stable only in solution. Hence none of them could be tested as catalysts in the transfer dehydrogenation reactions.

### 2.4 Catalytic Transfer Dehydrogenation

The pincer complexes **1**, **2**, and **3** were then tested for their catalytic activity in transfer dehydrogenation of alkanes, wherein the pre‐catalysts were activated using NaO*t*Bu to produce active Ir(I) species [14, 47, 50, 63, 64, 65]. The catalytic performance was evaluated with cyclooctane (COA) dehydrogenation as benchmark, using *tert*‐butylethylene (TBE) as the hydrogen acceptor as shown in Table 1.

**TABLE 1:** Ir catalyzed COA/TBE transfer dehydrogenation.

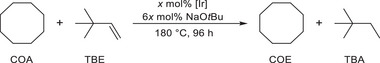			
Pre‐catalyst	[Ir] loading (mol %)	Total COA Conversion (%)	TON
1	0.1	63	630
1^a^	0.033	47	1410
1^b^	0.017	40	2286
2	0.1	25	250
3	0.1	52	520

^a^ Reaction conditions: Cyclooctane (2.2 mmol), TBE (2.2 mmol), [Ir] (2.2 µmol), base (13.2 µmol).

^b^ Cyclooctane (2.2 mmol), TBE (2.2 mmol), [Ir] (0.73 µmol), base (4.38 µmol).

^c^ Cyclooctane (2.2 mmol), TBE (2.2 mmol), [Ir] (0.5 µmol), base (3 µmol). Conversions were determined by GCMS analysis using mesitylene as internal standard, and TONs were calculated based on COA conversions.

Complexes **1** and **3** work well as pre‐catalysts, resulting in 63% and 52% conversion of the cyclooctane into cyclooctene, respectively. Similar behavior of **1** and **3** as pre‐catalysts can be expected as the pincer backbone is identical, thus likely producing the same catalytically active species in the presence of NaO*t*Bu. However, complex **2** led to only to 25% conversion of the substrate. In all reactions, the conversion of TBE was comparative to that of COA, and no formation of cyclooctadiene or cyclooctatriene was observed. The catalytic reactions were also run using lower catalyst loadings with complex **1**, from the initial catalyst loading of 0.1 mol%, to 0.033 mol% and to 0.017 mol%. All conditions produced reasonably good conversions of COA, and a maximum TON of 2286 was reached with 0.017 mol% loading. When compared to (POCN)Ir‐pincer complexes for the catalytic transfer dehydrogenation of cyclooctane, **1** performed superior to [(*^t^* ^Bu^POCN_Py_)Ir(H)(Cl)] [18] and [(*^t^* ^Bu^POCN_Imine_)Ir(H)(Cl)] [16], but has a lower activity than the [(*^t^* ^Bu^POCN_BQ_)Ir(H)(Cl)] [63] catalyst.

When *n*‐octane was used as substrate instead, 20 % TBE conversion and 8 % *n*‐octane conversion was observed using complexes **1** (Table 2). The reaction mixture contained *n*‐octene, *n*‐octadiene and *n*‐octatriene along with their isomers as products. This explains the difference in TBE and substrate conversions.

**TABLE 2:** Substrate scope for transfer dehydrogenation of alkanes using **1** as catalyst.

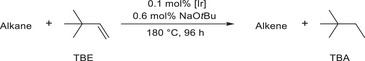			
Substrate	Product	TBE conversion (%)	Substrate conversion (%)
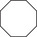	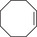	55	63
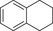	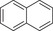	73	31
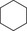	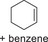	20	5
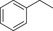	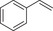	8	5
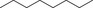	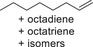	20	8
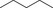	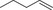	2	1

Reaction condition: Substrate (2.2 mmol), TBE (2.2 mmol), [Ir] (2.2 µmol), base (13.2 µmol). Conversions were determined by GCMS analysis using mesitylene as internal standard, and TONs were calculated based on substrate conversions.

Several other cyclic and linear alkane substrates were screened using **1**. Besides cyclooctane, tetralin worked well with 31% substrate conversion to yield naphthalene. For all other alkanes, a low conversion was obtained. Surprisingly, conversion for the transfer dehydrogenation of ethyl benzene was also only 5%.

Iridium pincer complexes with such unsymmetrical POCN ligands are also known to perform well in the transfer dehydrogenation of heterocycles to heteroarenes [18, 66]. Hence the same catalytic condition was used with tetrahydroquinoline as substrate. When **1** was used as catalyst, 54% TBE conversion and 29% substrate conversion to quinoline was obtained. On increasing the catalyst loading to 0.5 mol%, complete conversion of TBE was noted, along with 71% substrate conversion. Thus, several other unsaturated heterocycles were also screened using 0.5 mol% catalyst loading of **1,** as depicted in Table 3. All of the reaction mixtures of catalytic heterocycle dehydrogenation showed complete conversion of TBE to TBA, along with good substrate conversions. Indoline could be successfully dehydrogenated with a 100% conversion. Interestingly, the dehydrogenation of the challenging 3,5‐methylpiperidine to 3,5‐dimethylpyridine yielded a reasonable conversion of 54%. The catalytic activity of **1** for heterocylces is found to be comparable to that of reported [(*^t^* ^Bu^POCN_Py_)Ir(H)(Cl)] [18] and [(*^t^* ^Bu^POCN_BQ_)Ir(H)(Cl)] [63] catalysts.

**TABLE 3:** Substrate scope for transfer dehydrogenation of unsaturated heterocycles using **1** as catalyst.

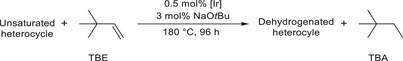			
Substrate	Product	TBE conversion (%)	Substrate conversion (%)
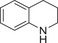	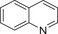	100	71
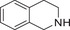	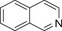	100	50
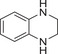	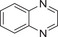	100	72
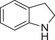	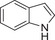	100	100
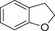	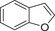	100	79
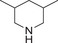	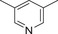	100	54

Reaction condition: Substrate (2.4 mmol), TBE (2.4 mmol), [Ir] (12 µmol), base (72 µmol). Conversions were determined by GCMS analysis using mesitylene as internal standard, and TONs were calculated based on substrate conversions.

## 3 Conclusion

In this report, a non‐symmetric *^t^* ^Bu^POCN_Triaz_ pincer ligand has been employed for the formation of a [(*^t^* ^Bu^POCN_Triaz_)Ir(H)(OAc)] (**1**) and the air and water stable complex [(*^t^* ^Bu^POCN_Triaz_)Ir(H)(acac)] (**3**). Attempts to metalate the ligand using [Ir(COD)(O_2_CCF_3_)]_2_ resulted in the tetrameric complex [(*^t^* ^Bu^POC)Ir(H)(O_2_CCF_3_)]_4_ (**2**), illustrating the influence of the anionic ligand in the formation of pincer complexes. Complexes **1** and **3** could be converted into the cyclometallated cationic complex (**6**) by using AgBF_4_. AgBF_4_ not only interacts with the anionic ligand, but also acts as an oxidizing agent, opening up reaction pathways, which might be based on PCET steps. On the other hand, we could also obtain the ionic species [(*^t^* ^Bu^POCN_Triaz_)Ir(H)(OAc)·B(C_6_F_5_)_3_] (**13**), by reaction of **1** with a Lewis acid. The pincer complexes **1** and **3** were further employed in the catalytic transfer dehydrogenations of alkanes as well as heterocycles. Substrate scope study with **1** reveals poor activity towards other alkanes, except for tetralin. However, the catalytic activity in the transfer dehydrogenation of unsaturated heterocycles with low catalyst loading could be demonstrated (0.5 mol%).

## 4 Experimental Section

### 4.1 Materials and Methods

Synthesis and reactions were carried out with a Schlenk line or inside a glovebox under argon atmosphere. Solvents were dried by the usual procedures, distilled, degassed and stored under argon atmosphere. All reagents required were procured from commercial sources. The metal precursors [(COD)Ir(OAc)]_2_ and [(COD)Ir(O_2_CCF_3_)]_2_ was synthesized as reported in literature [25], while [(COD)Ir(acac)] was commercially purchased.

All NMR spectra were recorded at a Bruker DPX 300, a Bruker Avance II 300 or a Bruker Avance 300 NMR spectrometer, at 298 K. The ^1^H NMR chemical shifts were referenced to residual CD_2_Cl_2_ at *δ* = 5.32 ppm or 1,2‐dichlorobenzene at *δ* = 6.93 ppm. The ^31^P{^1^H} NMR chemical shifts were referenced to external H_3_PO_4_ at *δ* = 0.00 ppm. Attenuated total reflection (ATR) IR spectra were recorded inside a glovebox at a Bruker ALPHA II spectrometer equipped with an ATR‐module (diamond).

Electrospray Ionization Mass Spectrometry (ESI‐MS) spectra were recorded by using an ADVION EXPRESSION CMS spectrometer (in typical ionization mode); acetonitrile was used as an eluent. Data analysis was carried out with ADVION DATA EXPRESS Version 6.0.11.3.

GC analysis was carried out using an AGILENT 7890B gas chromatograph (HP5 column, 30 m) with a flame‐ionization detector (FID) coupled to an EI‐MS AGILENT 5977B spectrometer with a triple‐axis detector. The instrument was equipped with an autoinjector Agilent G4513A (injection of 10 µL). The GC method starts with an oven temperature of 50°C (hold time 5 min) and a ramp from 50–200°C with 25°C/min followed by a hold time of 3 min at 200°C (total run‐time 14 min, solvent delay of 1.6 min). MS peaks were analyzed and compared with the library database of NIST MS Search 2.3. All EI‐MS spectra of the detected peaks were in good agreement with the library database of the detected substances. Quantification of products was done by comparison of the FID response to an internal standard and a calibration factor k that was determined by external calibration with known concentrations of product and internal standard.

### 4.2 Synthesis of the *^t^* ^Bu^POCN_Triaz_ Ligand Precursor

**Step 1**: In a round bottomed flask, 3‐ethynylphenol (1 g, 8.5 mmol,) and 1‐azidoadamantane (1.5 g, 8.5 mmol) were weighed, *t*BuOH (10 mL) and water (10 mL) were added and the solution was purged with argon. CuSO_4_ (40 mg, 0.25 mmol) and sodium ascorbate (198 mg, 1 mmol) were added and the resulting brown solution was heated to 60°C for 72 h. The reaction mixture was diluted with water and extracted with dichloromethane. The organic phase was dried over anhydrous magnesium sulphate, filtered and dried under vacuum to afford 3‐(1‐adamantan‐1‐yl)‐1*H*‐1,2,3‐triazol‐4‐yl)phenol as a white amorphous solid (1.41 g, 63%).

^1^H NMR (300 MHz, CD_2_Cl_2_) *δ* 7.86 (s, 1H, CH of triazole), 7.66 – 7.41 (m, 1H, Ar─H), 7.29 (m, 2H), 6.81 (m, 1H, Ar─H), 5.73 (s, 1H, OH), 2.28 (s, 9H, CH and CH_2_ of Ad), 1.83 (s, 6H, CH_2_ of Ad); ^13^C{^1^H} NMR (75 MHz, CD_2_Cl_2_) *δ* 156.8, 145.7, 133.0, 130.5, 118.1, 117.0, 115.2, 112.8, 60.2, 43.4, 36.2, 30.0.

**Step 2**: The obtained product (1.4 g, 4.8 mmol) was dissolved in 24 mL dry THF. NaH (232 mg, 5.8 mmol) was then added at 0°C and the solution was stirred for 15 min. Then a solution of ClP*t*Bu_2_ (0.9 mL, 4.8 mmol) in THF (4 mL) was added. The resulting reaction mixture was heated at 75°C for 20 h. The solution was filtered, and the leftover residue was washed twice using dichloromethane. The filtrate and washings were combined and volatiles were removed under vacuum, resulting in a solid. This was washed with *n*‐pentane and dried under vacuum to obtain the *^t^* ^Bu^POCN_Triaz_ ligand precursor as a white solid (2.1 g, 73%).

^1^H NMR (300 MHz, CD_2_Cl_2_) *δ* 7.86 (s, 1H, CH of triazole), 7.61 (q, *J*_H,H_ = 1.9 Hz, 1H, Ar─H), 7.40 (dt, *J*_H,H_ = 7.6, 1.2 Hz, 1H, Ar─H), 7.29 (t, *J*_H,H_ = 7.9 Hz, 1H, Ar─H), 7.17 – 7.10 (m, 1H, Ar─H), 2.28 (s, 9H, CH and CH_2_ of Ad), 1.82 (s, 6H, CH_2_ of Ad), 1.18 (d, *J*_P,H_ = 11.8 Hz, 18H, CH_3_ of P*t*Bu_2_); ^13^C{^1^H} NMR (75 MHz, CD2Cl2) *δ* 160.8 (d, *J*_C,P_ = 9 Hz), 146.6, 132.9, 130.1, 118.8, 118.0 (d, *J*_C,P_ = 11 Hz), 116.8, 115.7 (d, *J*_C,P_ = 10 Hz), 59.9, 43.3, 36.3, 35.9 (d, *J*_C,P_ = 25 Hz), 30.0, 27.5 (d, *J*_C,P_ = 15 Hz); ^31^P{^1^H} NMR (122 MHz, CD2Cl2) *δ* 154.8.

### 4.3 Synthesis of [(*^t^* ^Bu^POCN_Triaz_)Ir(H)(OAc)] (1)

The *^t^* ^Bu^POCN_Triaz_ ligand precursor (500 mg, 1.14 mmol), [Ir(COD)OAc]_2_ (410 mg, 0.57 mmol) and 35 mL of Toluene were added to a J‐Young flask. This reaction mixture was heated at 110°C for 20 h. The solution was filtered and the filtrate was concentrated to obtain a brown slurry. This was washed with *n‐*hexane and dried under vacuum to obtain a buff solid (730 mg, 92%).

^1^H NMR (300 MHz, CD_2_Cl_2_) *δ* 7.85 (s, 1H, CH of triazole), 6.97 (dd, *J*_H,H_ = 7.6, 0.9 Hz, 1H, Ar─H), 6.79 (td, *J*_H,H_ = 7.7, 0.9 Hz, 1H, Ar─H), 6.61 (d, *J*_H,H_ = 7.8 Hz, 1H, Ar─H), 2.32 (s, 9H, CH and CH_2_ of Ad), 1.89 (s, 3H, O_2_CCH_3_), 1.83 (s, 6H, CH_2_ of Ad), 1.40 – 1.21 (vt, *J*_P,H_ = 14 Hz,18H, CH_3_ of P*t*Bu_2_), – 30.51 (d, *J*_P,H_ = 22.5 Hz, 1H, Ir─H); ^13^C{^1^H} NMR (75 MHz, CD_2_Cl_2_) *δ* 186.3, 165.4 (d, *J*_C,P_ = 2 Hz), 155.8, 135.0, 134.1 (d, *J*_C,P_ = 1 Hz), 122.3, 115.5, 114.5, 108.9 (d, *J*_C,P_ = 10 Hz), 62.1, 43.1, 41.0 (d, *J*_C,P_ = 27 Hz), 39.5 (d, *J*_C,P_ = 31 Hz), 36.1, 30.1, 28.5 (d, *J*_C,P_ = 4 Hz), 27.1 (d, *J*_C,P_ = 4 Hz), 25.7; ^31^P{^1^H} NMR (122 MHz, CD_2_Cl_2_) *δ* 157.7; IR (ATR): ṽ = 2274 (s, H) cm^−1^; Elemental analysis for C_28_H_42_IrN_3_O_3_P: C, 48.61; H, 6.12; N, 6.07. Found: C, 49.278; H, 6.182; N, 6.028.

### 4.4 Synthesis of [(*^t^* ^Bu^POC)Ir(H)(O_2_CCF_3_)]_4_ (2)

The *^t^* ^Bu^POCN_Triaz_ ligand precursor (105 mg, 0.24 mmol), [Ir(COD)(O_2_CCF_3_)]_2_ (91 mg, 0.11 mmol) and 6.5 mL of Toluene were added to a J‐Young flask. The reaction mixture was heated at 110°C for 20 h. The solvent was removed by filtration, and the obtained solid residue was washed with Toluene and dried under vacuum to obtain a yellow solid (110 mg, 61%).

^1^H NMR (300 MHz, CD_2_Cl_2_) *δ* 7.76 (s, 1H, CH of triazole), 7.32 (d, *J*_H,H_ = 7.7 Hz, 1H, Ar─H), 7.20 (s, 1H, Ar─H), 6.70 (dd, *J*_H,H_ = 7.7, 1.4 Hz, 1H, Ar─H), 2.27 (s, 3H, CH of Ad), 2.21 (s, 6H, CH_2_ of Ad), 1.80 (s, 6H, CH_2_ of Ad), 1.19 (d, *J*_P,H_ = 14.1 Hz, 9H, CH_3_ of P*t*Bu_2_), 0.62 (d, *J*_P,H_ = 14.8 Hz, 9H, CH_3_ of P*t*Bu_2_), – 31.98 (d, *J*_P,H_ = 28.5 Hz, 1H, Ir─H); ^13^C{^1^H} NMR (75 MHz, CD_2_Cl_2_) *δ* 167.4 (d, *J*_C,P_ = 2 Hz), 151.4, 134.1, 131.2, 124.9, 122.0, 120.0, 110.3 (d, *J*_C,P_ = 10 Hz), 61.8, 42.9, 42.0 (d, *J*_C,P_ = 32 Hz), 38.2 (d, *J*_C,P_ = 40 Hz), 36.0, 30.0, 28.5 (d, *J*_C,P_ = 3 Hz), 27.2 (d, *J*_C,P_ = 3 Hz) (no peaks of O_2_CCF_3_ ligand observed in^13^C{^1^H} NMR spectrum); ^31^P{^1^H} NMR (122 MHz, CD_2_Cl_2_) *δ* 153.0; ^19^F{^1^H} NMR (282 MHz, CD_2_Cl_2_) *δ* −75.9; IR (ATR): ṽ = 2335 (s, H) cm; Elemental analysis for C_28_H_39_F_3_IrN_3_O_3_P: C, 45.09; H, 5.27; N, 5.63. Found: C, 45.877; H, 5.274; N, 5.394.

### 4.5 Synthesis of [(*^t^* ^Bu^POCN_Triaz_)Ir(H)(acac)] (3)

The *^t^* ^Bu^POCN_Triaz_ ligand precursor (174 mg, 0.39 mmol), [Ir(COD)(acac)] (143 mg, 0.36 mmol) and 15 mL of Toluene were added to a Schlenk flask. This reaction mixture was heated at 110°C for 20 h. The volatiles were removed under vacuum, the solid residue was washed with *n‐*pentane and dried under vacuum to obtain a yellow solid (226 mg, 86%).

^1^H NMR (300 MHz, CD_2_Cl_2_) *δ* 7.74 (s, 1H, CH of triazole), 6.95 (dd, *J*_H,H_ = 7.5, 0.9 Hz, 1H), 6.77 (td, *J*_H,H_ = 7.7, 0.9 Hz, 1H), 6.61 (d, *J*_H,H_ = 7.7 Hz, 1H), 5.23 (s, 1H, CH of acac), 2.26 (s, 9H, CH and CH_2_ of Ad), 1.94 (s, 3H, CH_3_ of acac), 1.82 (s, 6H), 1.54 (s, 3H, CH_3_ of acac), 1.30 (d, *J*_P,H_ = 14 Hz, 9H, CH_3_ of P*t*Bu_2_), 1.25 (d, *J*_P,H_ = 14 Hz, 9H, CH_3_ of P*t*Bu_2_), – 26.06 (d, *J*_P,H_ = 23.8 Hz, 1H, Ir─H); ^13^C{^1^H} NMR (75 MHz, CD_2_Cl_2_) *δ* 186.8, 184.5, 165.8, 155.5, 139.2 (d, *J*_C,P_ = 5 Hz), 135.0 (d, *J*_C,P_ = 1 Hz), 122.0, 115.4, 114.2, 108.4 (d, *J*_C,P_ = 10 Hz), 99.9, 61.4, 43.0, 41.1 (d, *J*_C,P_ = 25 Hz), 39.7 (d, *J*_C,P_ = 32 Hz), 36.1, 30.0, 28.2 (d, *J*_C,P_ = 4 Hz), 28.1, 28.0, 27.9 (d, *J*_C,P_ = 4 Hz); ^31^P{^1^H} NMR (122 MHz, CD_2_Cl_2_) *δ* 156.7; IR (ATR): ṽ = 2256 (s, H) cm^−1^; Elemental analysis for C_31_H_45_IrN_3_O_3_P: C, 50.94; H, 6.21; N, 5.75. Found: C, 51.605; H, 6.368; N, 5.676.

### 4.6 Formation of [(*^t^* ^Bu^POCN_Triaz_)Ir(CO)] (4)

In a J‐Young NMR tube, to a solution of **1** (10 mg, 14.5 µmol) or **3** (10 mg, 13.6 µmol) in 0.6 Toluene‐*d_8_* was added NaO*t*Bu (1.6 mg, 16.6 µmol). The solution was degassed, and CO gas was added to obtain an orange solution. The product was not isolated.

^1^H NMR (300 MHz, CD_2_Cl_2_) *δ* 7.62 (s, 1H, CH of triazole), 6.94‐6.83 (m, 3H, Ar─H), 2.30 (s, 9H, CH and CH_2_ of Ad), 1.82 (s, 6H, CH_2_ of Ad), 1.37 (d, *J*_P,H_ = 14 Hz, 18H); ^13^C{^1^H} NMR (75 MHz, CD_2_Cl_2_) *δ* 197.4 (d, *J*_C,P_ = 3 Hz), 167.9, 167.2 (d, *J*_C,P_ = 5 Hz), 160.7, 140.7, 127.6, 115.2, 114.3, 110.3 (d, *J*_C,P_ = 11 Hz), 61.9, 43.0, 41.2 (d, *J*_C,P_ = 29 Hz), 36.0, 30.0, 28.5 (d, *J*_C,P_ = 5 Hz); ^31^P{^1^H} NMR (122 MHz, CD_2_Cl_2_) *δ* 184.2; IR (ATR): ṽ = 1934 (s, CO) cm^−1^.

### 4.7 Formation of [(*^t^* ^Bu^POC)Ir(CO)_2_] (5)

In a J‐Young NMR tube, to a solution of **2** (10 mg, 13.4 µmol) in 0.6 1,2‐Dichlorobenzene‐*d_4_* NaO*t*Bu (1.5 mg, 16.1 µmol) was added. The solution was degassed, and CO gas was added to obtain a yellow solution. The product was not isolated. The experiment was also performed on using ^13^CO.

^1^H NMR (300 MHz, 1,2‐Dichlorobenzene‐*d_4_*) *δ* 8.20 – 8.01 (m, 1H, Ar─H), 8.02 – 7.91 (m, 1H, Ar─H), 7.72 (s, 1H, CH of triazole), 7.38 (dd, *J*_H,H_ = 7.6 Hz, *J*_C,H_ = 1.4 Hz, 1H, Ar─H), 2.13 (s, 6H, CH_2_ of Ad), 2.05 (s, 3H, CH of Ad), 1.62 (s, 6H, CH_2_ of Ad), 1.24 (d, *J*_P,H_ = 15.0 Hz, 18H, CH_3_ of P*t*Bu_2_); ^13^C NMR (75 MHz, 1,2‐Dichlorobenzene‐*d_4_*) *δ* 171.8 (d, *J*_C,P_ = 8.4 Hz), 152.0 (d, *J*_C,P_ = 8.9 Hz), 146.6, 144.4, 125.4, 119.3, 115.9, 108.5 (d, *J*_C,P_ = 13.2 Hz), 59.2, 43.0, 40.7 (d, *J*_C,P_ = 25.8 Hz), 35.9, 29.7, 28.0 (d, *J*_C,P_ = 5 Hz); ^31^P{^1^H} NMR (122 MHz, 1,2‐Dichlorobenzene‐*d_4_*) *δ* 187.8; IR (ATR): ṽ = 2037 (s, CO) cm^−1^, 1967 (s, CO) cm^−1^.

For **5**’: ^13^C{^1^H} NMR (75 MHz, 1,2‐Dichlorobenzene‐*d_4_*) *δ* 187.2 (dd, *J*_C,P_ = 8.2 Hz, *J*_C,C_ = 3.8 Hz, CO), 186.8 (dd, *J*_C,P_ = 105 Hz, *J*_C,C_ = 3.8 Hz, CO); ^31^P{^1^H} NMR (122 MHz, 1,2‐Dichlorobenzene‐*d_4_*) *δ* 187.8 (dd, *J*_C,P(trans)_ = 105 Hz, *J*_C,P(cis)_ = 8.2 Hz).

### 4.8 Formation of **6**

In a J‐Young NMR tube a solution of **1** (10 mg, 14.5 µmol) in 0.6 mL CD_2_Cl_2_ was treated with silver tetrafluoroborate (5.65 mg, 29 µmol). A green solution was obtained along with formation of gray precipitate. This solution was analyzed by NMR spectroscopy. Attempts to isolate the product failed.

^1^H NMR (300 MHz, CD_2_Cl_2_) *δ* 8.24 (s, 1H, CH of triazole), 7.38 (d, *J*_H,H_ = 8.1 Hz, 1H, Ar─H), 7.17 (t, *J*_H,H_ = 7.7 Hz, 1H, Ar─H), 6.97 (d, *J*_H,H_ = 7.8 Hz, 1H, Ar─H), 2.31 (s, 9H, CH and CH_2_ of Ad),1.97 (m, 1H, Ir─CH_2_), 1.84 (s, 3H, CH_2_ of Ad), 1.42 (d, *J*_P,H_ = 16.6 Hz, 9H, CH_3_ of P*t*Bu_2_), 1.39 (d, *J*_P,H_ = 18 Hz, 3H, CH_3_ of cyclometalated‐*t*Bu), 1.32 (m,1H, Ir─CH_2_) 1.02 (d, *J*_P,H_ = 14.4 Hz, 3H, CH_3_ of cyclometalated‐*t*Bu); ^13^C NMR peaks (from ^1^H,^13^C‐HSQC NMR and ^1^H,^13^C‐HMBC NMR) *δ* 163.6, 153.7, 135.3, 132.8, 123.6, 118.0, 114.3, 111.6, 65.0 (d, *J*_C,P_ = 32 Hz), 63.5, 42.5, 36.9 (d, *J*_C,P_ = 22 Hz), 35.3, 29.5, 27.4 (d, *J*_C,P_ = 2 Hz), 26.2 (d, *J*_C,P_ = 4 Hz), 25.8 (d, *J*_C,P_ = 2 Hz), −5.1 (d, *J*_C,P_ = 27 Hz),^31^P{^1^H} NMR (122 MHz, CD_2_Cl_2_) *δ* 86.7; ^19^F{^1^H} NMR (282 MHz, CD_2_Cl_2_) *δ* −151.4; ^11^B{^1^H} NMR (96 MHz, CD_2_Cl_2_) *δ* −1.0; MS (ESI‐MS, positive mode): for [M + 2 MeCN]^+^Calcd.: *m*/*z* = 712.27, Found. *m*/*z* = 712.5.

### 4.9 Formation of **7**

In a J‐Young NMR tube, to a solution of 1 (10 mg, 14.5 µmol) in 0.6 mL CD_2_Cl_2_ was added silver tetrafluoroborate (5.65 mg, 29 µmol). The resulting orange solution was degassed and CO was added. The product was not isolated.

^1^H NMR (300 MHz, CD_2_Cl_2_) *δ* 8.26 (s, 1H, CH of triazole), 7.55 – 7.50 (m, 1H, Ar─H), 7.41 – 7.33 (m, 1H, Ar─H), 7.17 (d, *J*_H,H_ = 7.8 Hz, 1H, Ar─H), 2.34 (s, 6H, Ad─H), 2.32 (s, 3H, Ad─H), 1.84 (s, 3H, Ad─H), 1.76‐1.65 (m, 1H, Ir─CH_2_)1.46 (d, *J*_P,H_ = 18.5 Hz, 9H, CH_3_ of P*t*Bu), 1.43 (d, *J*_P,H_ = 15.7 Hz, 3H, CH_3_ of cyclometalated‐*t*Bu), 1.20 (d, *J*_P,H_ = 16.9 Hz, 3H, CH_3_ of cyclometalated‐*t*Bu), 0.47 – 0.37 (m, 1H, Ir─CH_2_); ^13^C{^1^H} NMR (75 MHz, CD_2_Cl_2_) *δ* 161.1, 157.6, 138.4, 135.7, 129.3, 119.4, 118.6, 113.4 (d, *J*_C,P_ = 10.2 Hz), 64.2, 63.1, 42.9, 35.8, 30.0, 29.3 (d, *J*_C,P_ = 4 Hz), 27.6 (d, *J*_C,P_ = 3 Hz), 25.3 (d, *J*_C,P_ = 3 Hz), 6.8 (d, *J*_C,P_ = 27 Hz); ^31^P{^1^H} NMR (122 MHz, CD_2_Cl_2_) *δ* 96.0; ^19^F{^1^H} NMR (282 MHz, CD_2_Cl_2_) *δ* ‐151.8; ^11^B{^1^H} NMR (96 MHz, CD_2_Cl_2_) *δ* ‐1.0; IR (ATR): ṽ = 2069 (s, CO) cm^−1^, 2031 (s, CO) cm^−1^.

For **7’**: ^13^C{^1^H} NMR (75 MHz, CD_2_Cl_2_) *δ* 170.4 (dd, *J*_C,C_ = 2.4 Hz, *J*_C,P_ = 1.0 Hz CO), 161.19 (d, *J*_C,C_ = 2.4 Hz, CO); ^31^P{^1^H} NMR (122 MHz, CD_2_Cl_2_) *δ* 96.0 (m); MS (ESI‐MS, positive mode): for [M(^13^CO)_2_]^+^ Calcd.: *m/z* = 688.22, Found. *m/z* = 688.3, for [M(^13^CO) + MeCN]^+^ Calcd.: *m/z* = 700.25, Found. *m/z* = 700.3.

### 4.10 Formation of **8**

In a J‐Young NMR tube, to a solution of **1** (10 mg, 14.5 µmol) in 0.6 mL CD_2_Cl_2_ silver tetrafluoroborate (5.65 mg, 29 µmol) was added. To the resulting green solution 2,2′‐bipyridine (2.3 mg, 14.5 µmol) was added. The solution was filtered over celite, and the filtrate was dried. The resulting solid was washed with ethyl acetate and dried under vacuum to obtain a brown solid.

^1^H NMR (300 MHz, CD_2_Cl_2_) *δ* 9.46 (d, *J*_H,H_ = 5.5 Hz, 1H, bipy‐H), 8.51 (d, *J*_H,H_ = 8.2 Hz, 1H, bipy‐H), 8.34 (m, *J*_H,H_ = 7.9, 1.5 Hz, 2H, two overlapping peaks of bipy‐H), 8.02 (s, 1H, CH of triazole), 8.01 – 7.91 (m, 2H, two overlapping peaks of bipy‐H), 7.86 (ddd, *J*_H,H_ = 7.3, 5.5, 1.3 Hz, 1H, bipy‐H), 7.48 (dd, *J*_H,H_ = 7.3, 1.0 Hz, 1H, Ar─H), 7.37 – 7.24 (m, 2H, two overlapping peaks of bipy‐H and Ar─H), 7.18 – 7.08 (m, 1H, Ar─H), 2.14 (s, 3H, Ad─H), 1.98 (d, J = 2.9 Hz, 5H, Ad─H), 1.68 (m, 6H, Ad─H), 1.37 (d, *J*_P,H_ = 18 Hz, 3H, CH_3_ of cyclometalated‐*t*Bu), 1.32 (m, 1H, Ir─CH_2_, overlapping with peak at 1.37 ppm) 1.09 (d, J = 14 Hz, 3H, CH_3_ of cyclometalated‐*t*Bu), 1.00 – 0.93 (m, 1H, Ir─CH_2_), 0.83 (d, *J*_P,H_ = 15.8 Hz, 9H, CH_3_ of P*t*Bu); ^13^C{^1^H} NMR (75 MHz, CD_2_Cl_2_) *δ* 164.3, 163.9, 157.8, 155.9, 154.6, 151.7, 150.0, 139.7, 138.8, 127.7, 127.4, 124.4, 123.9, 117.1, 113.1, 110.8 (d, *J*_C,P_ = 9.4 Hz), 65.0 (d, *J*_C,P_ = 32 Hz), 62.5, 42.6, 35.8 (d, *J*_C,P_ = 19 Hz), 35.8, 29.8, 28.7 (d, *J*_C,P_ = 4 Hz), 25.4 (d, *J*_C,P_ = 3 Hz), 25.1 (d, *J*_C,P_ = 4 Hz), – 2.4 (d, *J*_C,P_ = 29 Hz); ^31^P{^1^H} NMR (122 MHz, CD_2_Cl_2_) *δ* 89.7; ^19^F{^1^H} NMR (282 MHz, CD_2_Cl_2_) *δ* ‐152.6; ^11^B{^1^H} NMR (96 MHz, CD_2_Cl_2_) *δ* ‐1.1; MS (ESI‐MS, positive mode): for [M]^+^Calcd.: *m/z* = 786.29, Found. *m/z* = 786.3.

### 4.11 Formation of [(*^t^* ^Bu^POCN_Triaz_)Ir(H)(acac)·AgBF_4_] (9)

In a J‐Young NMR tube, to a solution of **3** (10 mg, 13.6 µmol) in 0.6 mL CD_2_Cl_2_ silver tetrafluoroborate (2.65 mg, 13.6 µmol) was added. A yellow solution was obtained along with formation of a gray precipitate. The solution was analyzed by NMR spectroscopy. Attempts to isolate the product failed.

^1^H NMR (300 MHz, CD_2_Cl_2_) *δ* 7.87 (s, 1H, CH of triazole), 7.02 (d, *J* = 7.5 Hz, 1H, Ar─H), 6.91 (t, *J* = 7.7 Hz, 1H, Ar─H), 6.75 (d, *J* = 7.7 Hz, 1H, Ar─H), 2.32 (s, 3H, CH_2_ of Ad), 2.25 (s, 6H, CH_2_ of Ad), 1.83 (s, 6H, CH_2_ of Ad), 1.38 (d, *J*_P,H_ = 14.9 Hz, 9 H, CH_3_ of P*t*Bu_2_), 1.25 (d, *J*_P,H_ = 14.7 Hz, 9 H, CH_3_ of P*t*Bu_2_), – 29.58 (d, *J*_P,H_ = 22.4 Hz, 1H, Ir─H); ^13^C NMR peaks (from ^1^H,^13^C‐HSQC NMR and ^1^H,^13^C‐HMBC NMR) *δ* 165.5, 156.0, 129.4, 124.4, 116.2, 115.5, 110.3, 62.8, 43.0, 41.7, 40.3, 35.8, 29.9, 28.0 (d, *J*_C,P_ = 4 Hz), 27.5 (d, *J*_C,P_ = 3 Hz); ^31^P{^1^H} NMR (122 MHz, CD_2_Cl_2_) *δ* 154.7, ^19^F{^1^H} NMR (282 MHz, CD_2_Cl_2_) *δ* ‐151.9; ^11^B{^1^H} NMR (96 MHz, CD_2_Cl_2_) *δ* ‐1.1; MS (ESI‐MS, positive mode): for [M + MeCN]^+^ Calcd.: *m/z* = 673.26, Found. *m/z* = 673.5, for [M + 2 MeCN]^+^ Calcd.: *m/z* = 714.29, Found. *m/z* = 714.5.

### 4.12 Formation of [(*^t^* ^Bu^POCN_Triaz_)Ir(H)(CO)_2_][BF_4_] (10)

In a J‐Young NMR tube, to a solution of **3** (10 mg, 13.6 µmol) in 0.6 mL CD_2_Cl_2_ was added silver tetrafluoroborate (2.65 mg, 13.6 µmol), to obtain complex **9**. This reaction mixture was degassed and CO gas was added resulting in a light‐yellow solution. The product was not isolated.

^1^H NMR (300 MHz, CD_2_Cl_2_) *δ* 8.33 (s, 1H, CH of triazole), 7.45 (d, *J*_H,H_ = 7.4 Hz, 1H, Ar─H), 7.29 (t, *J*_H,H_ = 7.9 Hz, 1H, Ar─H), 7.06 (d, *J*_H,H_ = 8.0 Hz, 1H, Ar─H), 2.34 (s, 9H, CH and CH_2_ of Ad), 1.85 (s, 6H, CH and CH_2_ of Ad), 1.52 (d, *J*_P,H_ = 16.6 Hz, 9H, CH_3_ of P*t*Bu_2_), 1.36 (d, *J*_P,H_ = 16.6 Hz, 9H, CH_3_ of P*t*Bu_2_), – 10.04 (d, *J*_P,H_ = 16.8 Hz, 1H, Ir─H); ^13^C{^1^H} NMR (75 MHz, CD_2_Cl_2_) *δ* 167.9 (Ir─CO), 163.0, 158.9, 136.0, 133.7, 129.4, 119.1, 118.6, 112.9 (d, *J*_C,P_ = 10.6 Hz), 64.4, 43.2 (d, *J*_C,P_ = 29.8 Hz), 42.9, 40.2 (d, *J*_C,P_ = 28.2 Hz), 35.8, 30.1, 28.4 (d, *J*_C,P_ = 3.9 Hz), 27.6 (d, *J*_C,P_ = 3.5 Hz); ^31^P{^1^H} NMR (122 MHz, CD_2_Cl_2_) *δ* 170.5; ^19^F{^1^H} NMR (282 MHz, CD_2_Cl_2_) *δ* ‐152.4; ^11^B{^1^H} NMR (96 MHz, CD_2_Cl_2_) *δ* ‐1.0; IR (ATR): ṽ = 2112 (s, CO) cm^−1^, 2077 (s, CO) cm^−1^; MS (ESI‐MS, positive mode): for [M—CO + MeCN]^+^Calcd.: *m/z* = 701.23, Found. *m/z* = 701.8.

For **10’**: ^1^H NMR (300 MHz, CD_2_Cl_2_) *δ* – 10.03 (ddd, *J*_C,H_ (trans) = 55.9 Hz, *J*_P,H_ = 16.8 Hz, *J*_C,H_ (cis) = 5.6 Hz 1H, Ir‐H) (rest of the spectrum was identical to that of 7); ^13^C NMR (101 MHz, CD_2_Cl_2_) *δ* 167.5 (ddd, *J*_C,H_ (cis) = 5.4 Hz, *J*_C,C_ = 2.4 Hz, *J*_C,P_ = 1.2 Hz, CO), 166.2 (ddd, *J*_C,H_ (trans) = 55.9 Hz, *J*_C,C_ = 2.4 Hz, *J*_C,P_ = 3.6 Hz, CO); ^31^P{^1^H} NMR (122 MHz, CD_2_Cl_2_) *δ* 170.5 (dd, *J*_C,P_ = 3.6 Hz, 1.2 Hz).

### 4.13 Formation of [(*^t^* ^Bu^POCN_Triaz_)Ir(acac)][BF_4_] (11)

In a J‐Young NMR tube, to a solution of **3** (10 mg, 13.6 µmol) in 0.6 mL CD_2_Cl_2_ silver tetrafluoroborate (2.65 mg, 13.6 µmol) was added. A yellow solution was obtained along with formation of a gray precipitate. Formation of complex **10** was observed in the reaction mixture after 5 days at room temperature. Attempts to isolate the product failed.

^1^H NMR (300 MHz, CD_2_Cl_2_) *δ* 7.90 (s, 1H, CH of triazole), 7.22 (d, *J*_H, H_ = 7.1 Hz, 1H, Ar─H), 7.11 – 6.95 (m, 1H, Ar─H), 6.91 (d, *J*_H, H_ = 8.5 Hz, 1H, Ar─H), 5.56 (s, 1H, CH of acac), 2.30 (s, 9H, CH and CH_2_ of Ad), 2.24 (s, 3H, CH_3_ of acac), 1.83 (s, 6H, CH_2_ of Ad), 1.53 (s, 3H, CH_3_ of acac), 1.36 (d, *J*_P, H_ = 14 Hz, 9H, CH_3_ of P*t*Bu_2_), 1.19 (d, *J*_P, H_ = 14 Hz, 9H, CH_3_ of P*t*Bu_2_); ^13^C{^1^H} NMR (75 MHz, CD_2_Cl_2_) *δ* 190.3, 185.1, 155.5, 137.4, 127.1, 116.5, 116.4, 110.7 (d, *J*_C,P_ = 9.9 Hz), 103.0, 62.8, 42.8, 42.1 (d, *J*_C,P_ = 11.4 Hz), 36.1, 30.1, 28.5 (d, *J*_C,P_ = 4.2 Hz), 28.1 (d, *J*_C,P_ = 3.9 Hz), 27.8, 25.8 (the quaternerary Ir─C could not be observed in the ^13^C{^1^H}, ^13^C‐HSQC and ^1^H, ^13^C‐HMBC NMR spectra); ^31^P{^1^H} NMR (122 MHz, CD_2_Cl_2_) *δ* 133.3; ^19^F{^1^H} NMR (282 MHz, CD_2_Cl_2_) *δ* ‐151.2; MS (ESI‐MS, positive mode): for [M + MeCN]^+^ Calcd.: *m/z* = 771.30, Found. *m/z* = 771.5.

### 4.14 Formation of [(*^t^* ^Bu^POCN_Triaz_)Ir(acac)(CO)][BF_4_] (12)

The J‐Young NMR tube containing complex **11** was degassed and CO gas was added. This led to formation of complex **12**. The product was not isolated.

^1^H NMR (300 MHz, CD_2_Cl_2_) *δ* 8.27 (s, 1H, CH of triazole), 7.43 (d, *J*_H,H_ = 7.6 Hz, 1H, Ar─H), 7.30 (t, *J*_H,H_ = 7.8 Hz, 1H, Ar─H), 7.05 (d, *J*_H,H_ = 8.0 Hz, 1H, Ar─H), 5.62 (s, 1H, CH of acac), 2.44 – 2.11 (m, 9H, CH and CH_2_ of Ad; 3H, overlapping CH_3_ of acac), 1.84 (s, 6H, CH_2_ of Ad), 1.59 (d, 9H, *J*_P,H_ = 17.4 Hz, CH_3_ of P*t*Bu_2_; 3H, overlapping CH_3_ of acac), 1.27 (d, *J*_P,H_ = 15.7 Hz, 9H, CH_3_ of P*t*Bu_2_); ^13^C{^1^H} NMR (75 MHz, CD_2_Cl_2_) *δ* 193.3, 184.4, 177.0, 163.8, 155.1, 135.5, 129.8, 123.5, 119.2, 118.3, 113.0 (d, *J*_C,P_ = 9.3 Hz), 103.2, 64.1, 43.6 (d, *J*_C,P_ = 22.5 Hz), 42.8, 41.4 (d, *J*_C,P_ = 27.7 Hz), 35.9, 30.1, 29.0 (d, *J*_C,P_ = 3.7 Hz), 28.3 (d, *J*_C,P_ = 3.1 Hz), 27.9, 26.6; ^31^P{^1^H} NMR (122 MHz, CD_2_Cl_2_) *δ* 149.8; ^19^F{^1^H} NMR (282 MHz, CD_2_Cl_2_) *δ* ‐152.0; ^11^B{^1^H} NMR (96 MHz, CD_2_Cl_2_) *δ* ‐1.1; IR (ATR): ṽ = 2061 (s, CO) cm^−1^; MS (ESI‐MS, positive mode): for [M]^+^ Calcd.: *m/z* = 758.27, Found. *m/z* = 578.3.

### 4.15 Formation of [(*^t^* ^Bu^POCN_Triaz_)Ir(H)(OAc)·B(C_6_F_5_)_3_] (13)

In a J‐Young NMR tube, to a solution of **1** (10 mg, 14.5 µmol) in 0.6 mL CD_2_Cl_2_ tris(pentafluorophenyl)borane (7.4 mg, 14.5 µmol) was added resulting in a yellow solution. The product was not isolated.

^1^H NMR (300 MHz, CD_2_Cl_2_) *δ* 7.66 (s, 1H, CH of triazole), 7.04 – 6.82 (m, 3H, Ar─H), 2.18 (s, CH_3_ of [B(OAc)(C_6_F_5_)_3_]), 1.88 (m, 9H, CH and CH_2_ of Ad), 1.68 (s, 6H, CH_2_ of Ad), 1.54 (d, *J*_P,H_ = 15.1 Hz, 9 H, CH_3_ of P*t*Bu_2_), 1.33 (d, *J*_P,H_ = 15.2 Hz, 9 H, CH_3_ of P*t*Bu_2_), – 43.02 (s, broad, 1H, Ir─H); ^13^C{^1^H} NMR peaks (from ^1^H,^13^C‐HSQC NMR and ^1^H,^13^C‐HMBC NMR) *δ* 166.4, 156.7, 137.2, 126.2, 116.2, 110.9, 62.7, 42.6, 41.4 (d, *J*_C,P_ = 20 Hz), 40.4 (d, *J*_C,P_ = 20 Hz) 35.7, 29.7, 27.9 (d, *J*_C,P_ = 3.2 Hz), 27.1 (d, *J*_C,P_ = 3.2 Hz); ^31^P{^1^H} NMR (122 MHz, CD_2_Cl_2_) *δ* 157.0; ^19^F{^1^H} NMR (282 MHz, CD_2_Cl_2_) *δ*; – 134.6 (d, *J*_F,F_ = 20.6 Hz), – 160.0 (t, *J*_F,F_ = 20.2 Hz), – 166.0 (t, *J*_F,F_ = 18.7 Hz); ^11^B{^1^H} NMR (96 MHz, CD_2_Cl_2_) *δ* ‐1.6 (broad); MS (ESI‐MS, positive mode): for [M + MeCN]^+^ Calcd.: *m/z* = 673.26, Found. *m/z* = 673.6, for [M + 2 MeCN]^+^ Calcd.: *m/z* = 714.29, Found. *m/z* = 714.7; MS (ESI‐MS, negative mode): for [M]^−^ Calcd.: *m/z* = 571.00, Found. *m/z* = 571.0.

### 4.16 Formation of [(*^t^* ^Bu^POCN_Triaz_)Ir(H)(2,2′‐bipyridine)][B(OAc)(C_6_F_5_)_3_] (14)

In a J‐Young NMR tube, to a solution of **1** (10 mg, 14.5 µmol) in 0.6 mL CD_2_Cl_2_ tris(pentafluorophenyl)borane (7.4 mg, 14.5 µmol) was added. To the resulting yellow solution was added 2,2′‐bipyridine (2.3 mg, 14.5 µmol). The product was not isolated.

^1^H NMR (300 MHz, CD_2_Cl_2_) *δ* 9.38 (d, *J*_H,H_ = 5.1 Hz, 1H, bipy‐H), 8.39 (d, *J*_H,H_ = 8.2 Hz, 1H, bipy‐H), 8.31 – 8.15 (m, 2H, bipy‐H), 7.91 (td, *J*_H,H_ = 8.0, 1.5 Hz, 1H, bipy‐H), 7.75 (s, 1H, CH of triazole), 7.69 (d, *J*_H,H_ = 5.3 Hz, 1H, bipy‐H), 7.65 – 7.57 (m, 1H, bipy‐H), 7.30 – 7.02 (m, 3H, overlapping bipy‐H and Ar─H), 6.90 (d, *J*_H,H_ = 7.6 Hz, 1H, Ar─H), 2.13 (s, 3H, CH_3_ of anion), 1.94 – 1.88 (m, 9H, CH and CH_2_ of Ad), 1.77 – 1.55 (m, 6H, CH_2_ of Ad), 1.35 (d, *J*_P,H_ = 14.6 Hz, 9H, CH_3_ of P*t*Bu_2_), 0.81 (d, *J*_P,H_ = 14.6 Hz, 9H, CH_3_ of P*t*Bu_2_), ‐20.36 (d, *J*_P,H_ = 25.2 Hz, 1H, Ir─H); ^13^C{^1^H} NMR (75 MHz, CD_2_Cl_2_) *δ* 172.9, 163.8, 156.8, 156.4, 156.2, 149.7, 141.7, 139.1, 138.9, 134.3, 127.7, 127.2, 124.9, 124.3, 123.9, 116.5, 115.6, 110.7 (d, *J*_C,P_ = 10.6 Hz), 62.4, 42.7, 42.9 (d, *J*_C,P_ = 25.4 Hz), 41.2 (d, *J*_C,P_ = 26.1 Hz), 35.8, 30.1, 29.8, 28.2, 27.9, 23.7; ^31^P{^1^H} NMR (122 MHz, CD_2_Cl_2_) *δ* 151.0; ^19^F{^1^H} NMR (282 MHz, CD_2_Cl_2_) *δ* – 133.3 to – 135.7 (m), – 163.2 (t, *J*_F,F_ = 20.3 Hz), – 167.3 (td, *J*_F,F_ = 23.5, 6.6 Hz); ^11^B{^1^H} NMR (96 MHz, CD_2_Cl_2_) *δ* – 5.1; MS (ESI‐MS, positive mode): for [M]^+^ Calcd.: *m*/*z* = 788.31, Found. *m*/*z* = 788.6; MS (ESI‐MS, negative mode): for [M]^−^ Calcd.: *m*/*z* = 571.00, Found. *m/z* = 571.2.

## Conflicts of Interest

The authors declare no conflicts of interest.

## Supporting information



Additional supporting information can be found online in the Supporting Information section.
Supporting File: Contains figures related to procedures and observations (S1 – S17), NMR spectra (S18 – S84) and X‐ray crystallographic data for new compounds CCDC 2468854 (Table S1) and CCDC 2531616 (Table S2). The crystallographic data can be obtained free of charge from The Cambridge Crystallographic Data Centre via www.ccdc.cam.ac.uk/structures. Additional references are cited within the Supporting Information [1, 2, 3, 4].

## Data Availability

The data are available in the Supporting Information of the manuscript.
