# Supplementary material for: Iridium Complexes of a Triazole‐Derived Pincer Ligand: Synthesis, Reactivity, and Transfer Dehydrogenation Catalysis
Source: Chemistry. 2026 Apr 1;32(22):e70909. doi: 10.1002/chem.70909 (PMC13250354; doi:10.1002/chem.70909)
Supplement: Supplementary file 1 — Additional supporting information can be found online in the Supporting Information section. Supporting File: Contains figures related to procedures and observations (S1 – S17), NMR spectra (S18 – S84) and X‐ray crystallographic data for new compounds CCDC 2468854 (Table S1) and CCDC 2531616 (Table S2). The crystallographic data can be obtained free of charge from The Cambridge Crystallographic Data Centre via www.ccdc.cam.ac.uk/structures. Additional references are cited within the Supporting Information [1, 2, 3, 4]. [file CHEM-32-e70909-s001.docx]

Supporting Information

**Iridium Pincer Complexes of a New Triazolophosphinite Pincer Ligand: Synthesis, Reactivity and Catalysis**

Jesvita Cardozo,^[a]^ Ouchan He,^[a]^ Aaron Prenzlow,^[a]^ Xinyang Peng,^[a]^ Kallol Ray^[a]^ and Thomas Braun*^[a]^

**Table of Contents**

[**1 Observation of by-products for Scheme 4** 2](#_Toc222486404)

[**1.1 ^1^H NMR Spectrum indicating fluorosilicates** 2](#_Toc222486405)

[**1.2 ^19^F{^1^H} NMR Spectra** 2](#_Toc222486406)

[**1.3 Reaction monitoring with ^31^P{^1^H} NMR Spectra** 3](#_Toc222486407)

[**2 Reaction monitoring for Scheme 5** 4](#_Toc222486408)

[**2.1 ^31^P{^1^H} NMR spectra** 4](#_Toc222486409)

[**2.2 ESI-MS spectra** 5](#_Toc222486410)

[**2.3 Observations for side product formation** 6](#_Toc222486411)

[**3 Low Temperature ^1^H NMR spectra for Complex 13** 9](#_Toc222486412)

[**4 Procedure for Alkane Transfer Dehydrogenation Reactions** 10](#_Toc222486413)

[**5 Proof of tetrameric structure of [(*^t^*^Bu^POC)Ir(O_2_CCF_3_)(H)]_4_ (2)** 11](#_Toc222486414)

[**5.1 IR spectra of 2** 11](#_Toc222486415)

[**5.2 ^1^H,^1^H-NOESY spectrum of 2** 12](#_Toc222486416)

[**5.3 Molecular Structure of 2** 12](#_Toc222486417)

[**6 Cyclic Voltammetry** 13](#_Toc222486418)

[**7 Reactivity of [(*^t^*^Bu^POCN_Triaz_)Ir(H)(O_2_CCH_3_)]** **(1) with other reagents** 14](#_Toc222486419)

[**8 Reactivity of [(*^t^*^Bu^POCN_Triaz_)Ir(H)(acac)]** **(3) with other reagents** 14](#_Toc222486420)

[**9 X-Ray Crystallographic Data** 15](#_Toc222486421)

[**10 NMR Spectra** 17](#_Toc222486422)

[**11 References** 50](#_Toc222486423)

**1 Observation of by-products for Scheme 4**

**1.1 ^1^H NMR Spectrum indicating fluorosilicates**


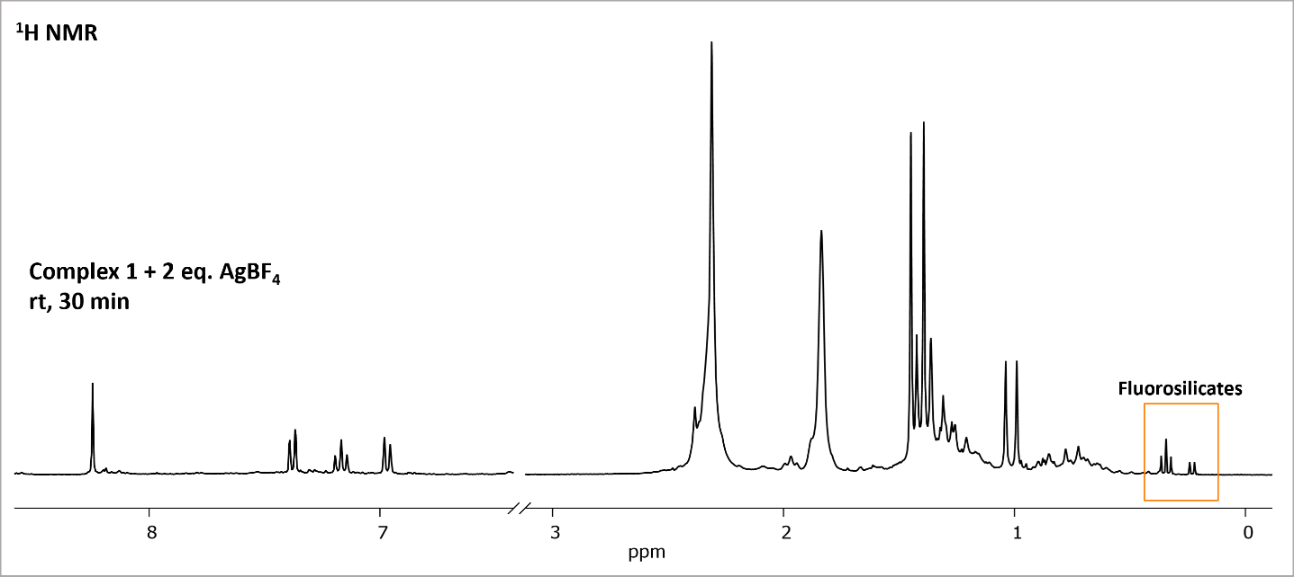


**Figure S1**. ^1^H NMR spectrum indicating formation of fluorosilicates.

**1.2 ^19^F{^1^H} NMR Spectra**


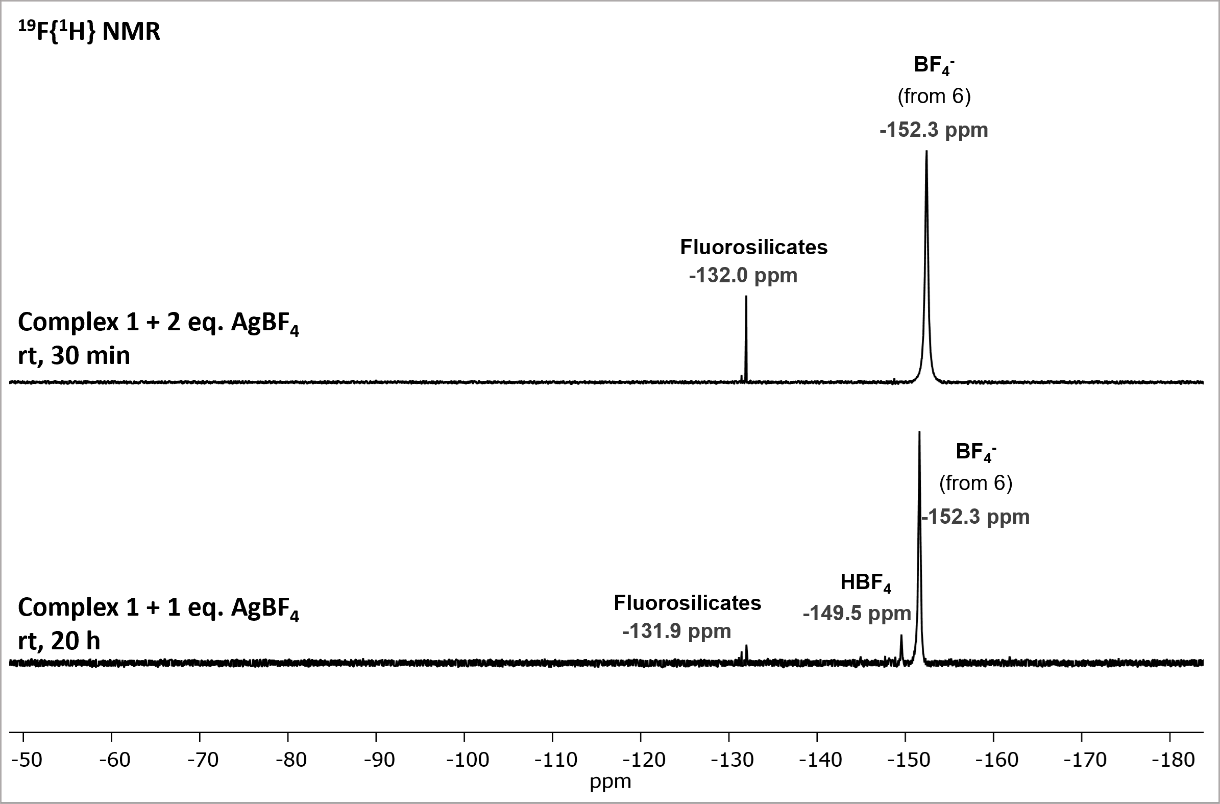


**Figure S2**. ^19^F{^1^H} NMR spectrum indicating formation of fluorosilicates.

**1.3 Reaction monitoring with ^31^P{^1^H} NMR Spectra**

When the reaction of **1** with 1 eq. of AgBF_4_ was monitored over 20 h, the ^31^P{^1^H} NMR spectra suggested formation of **6** whereas the ^1^H NMR spectrum was messy. Although the reaction was monitored for up to 20 h, no other intermediates were observed.

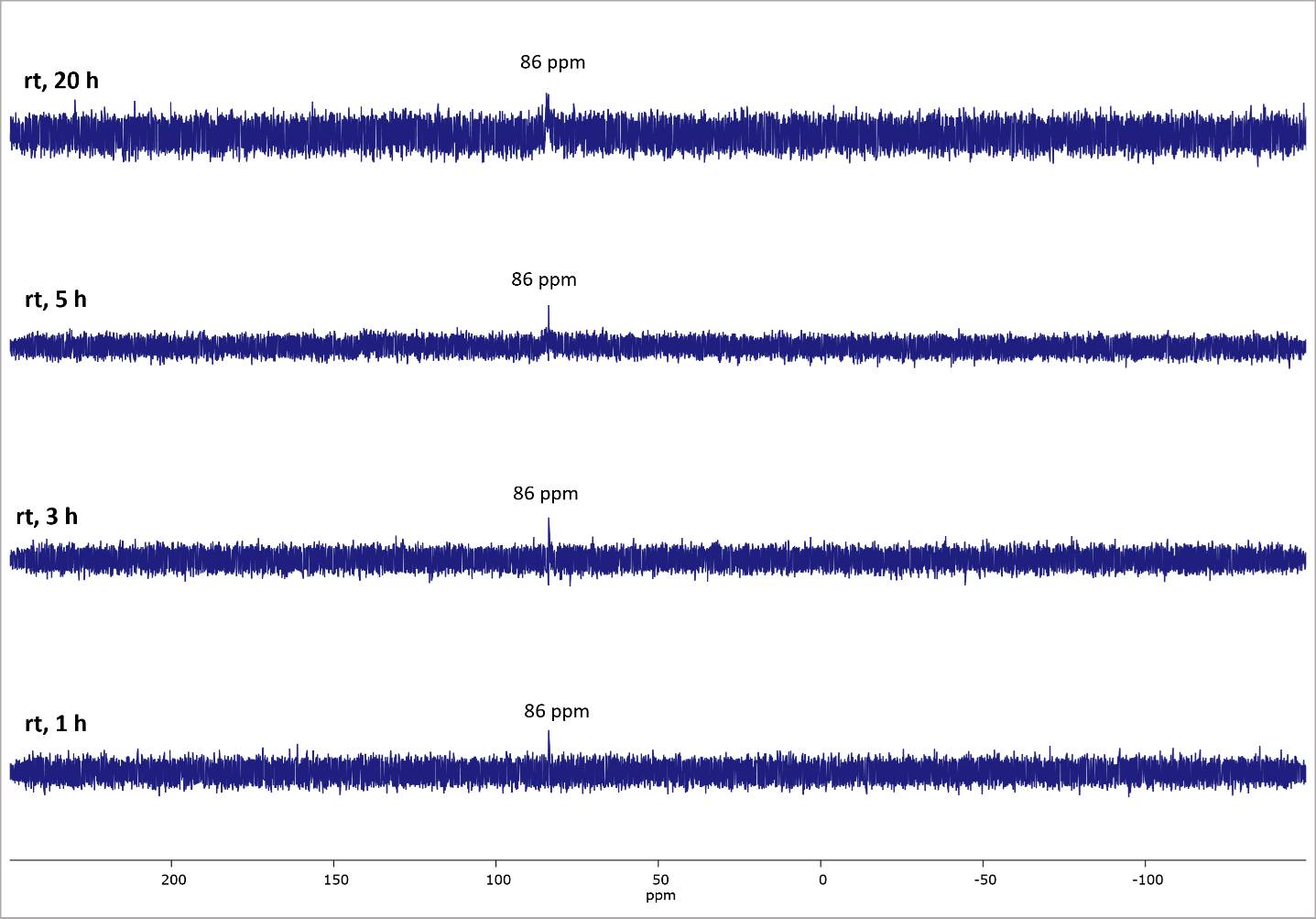


**Figure S3**. ^31^P{^1^H} spectra of reaction mixture of **1** with AgBF_4_ various times.

*All NMR spectra measured in CD_2_Cl_2_ at 300 MHz

**2 Reaction monitoring for Scheme 5**

**2.1 ^31^P{^1^H} NMR spectra**


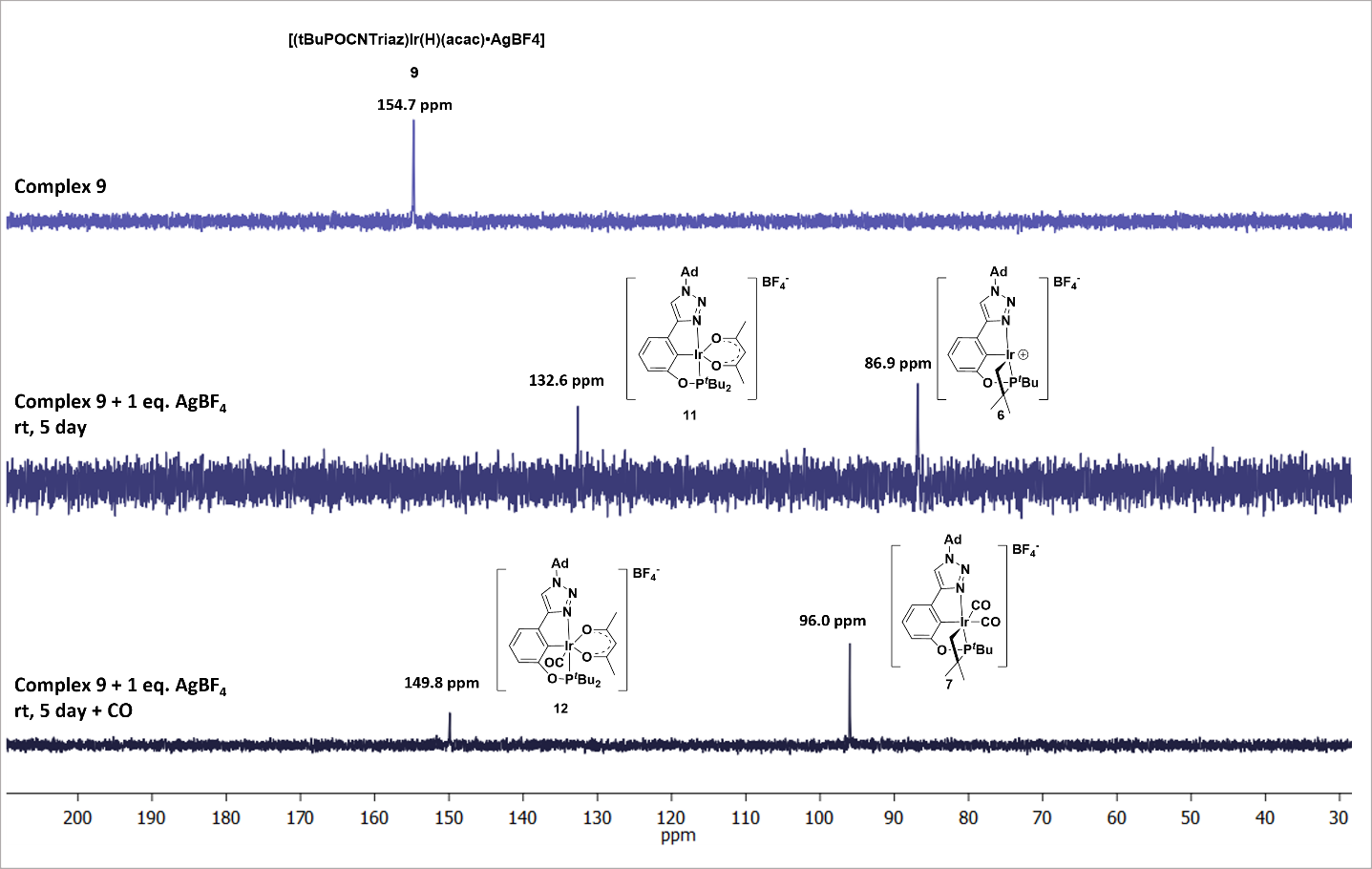


**Figure S4**. ^31^P{^1^H} NMR monitoring for reactivity of **9** with AgBF_4_.

*All NMR spectra measured in CD_2_Cl_2_ at 300 MHz

**2.2 ESI-MS spectra**

**
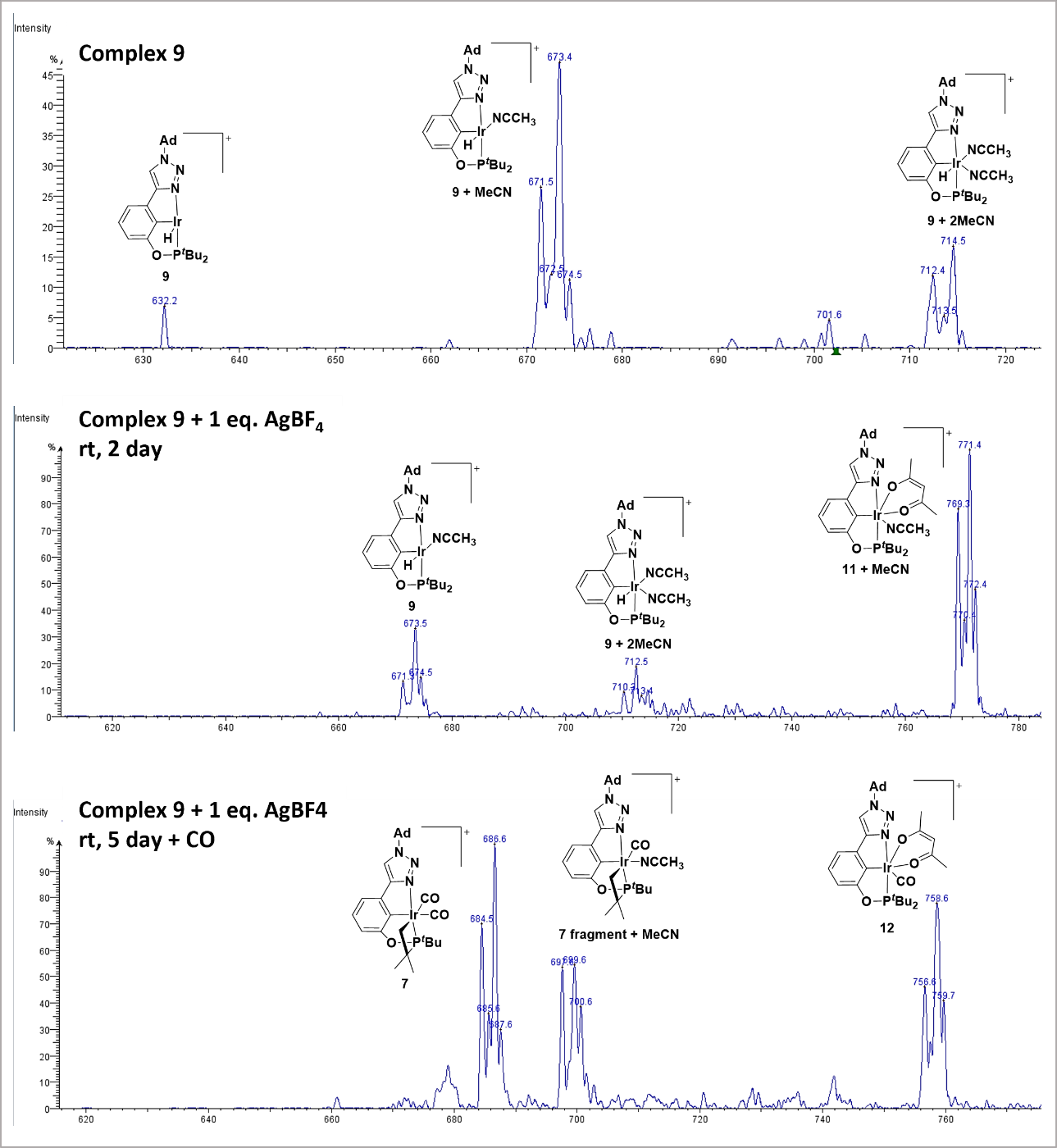
**

**Figure S5**. ESI-MS (positive mode) monitoring for reactivity of **9** with AgBF_4_.

**2.3 Observations for side product formation**

Formation of HBF_4_ and (acetylacetonato)difluoroboron have been observed in the following reaction of Scheme 5

**Formation of HBF_4_:**

Addition of 1 equivalent of AgBF_4_ to complex **9**, leads to disappearance of the silicone grease peak in ^1^H NMR spectrum and instead peaks indicating formation of fluorosilicates appear. This indicates formation of HBF_4_ in the reaction.

**
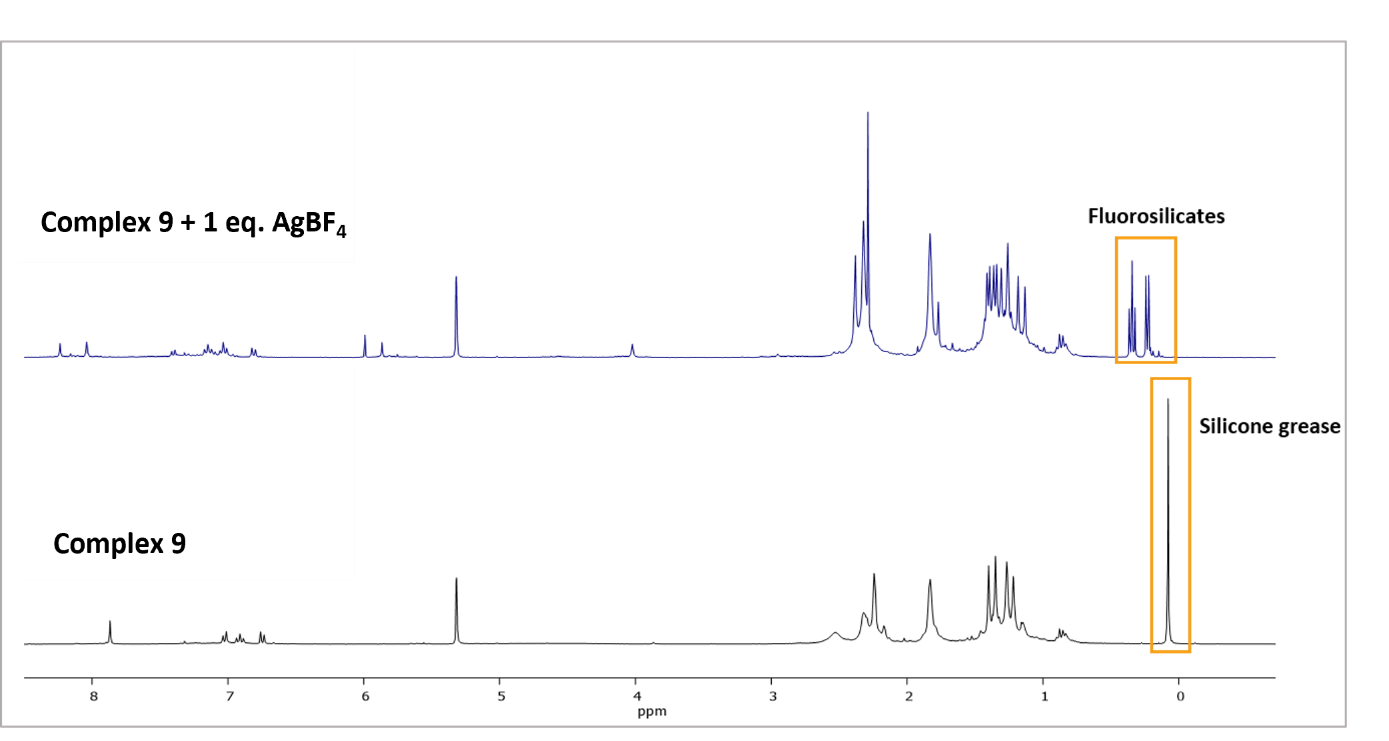
**

**Figure S6**. ^1^H NMR monitoring for reactivity of **9** with AgBF_4_.

*All NMR spectra measured in CD_2_Cl_2_ at 300 MHz

**Formation of (acetylacetonato)diflouorboron:**

The formation of complex **6** produces Hacac, which further reacts in a side reaction as follows:

**Side Reaction:**

**Test Reaction:**

**
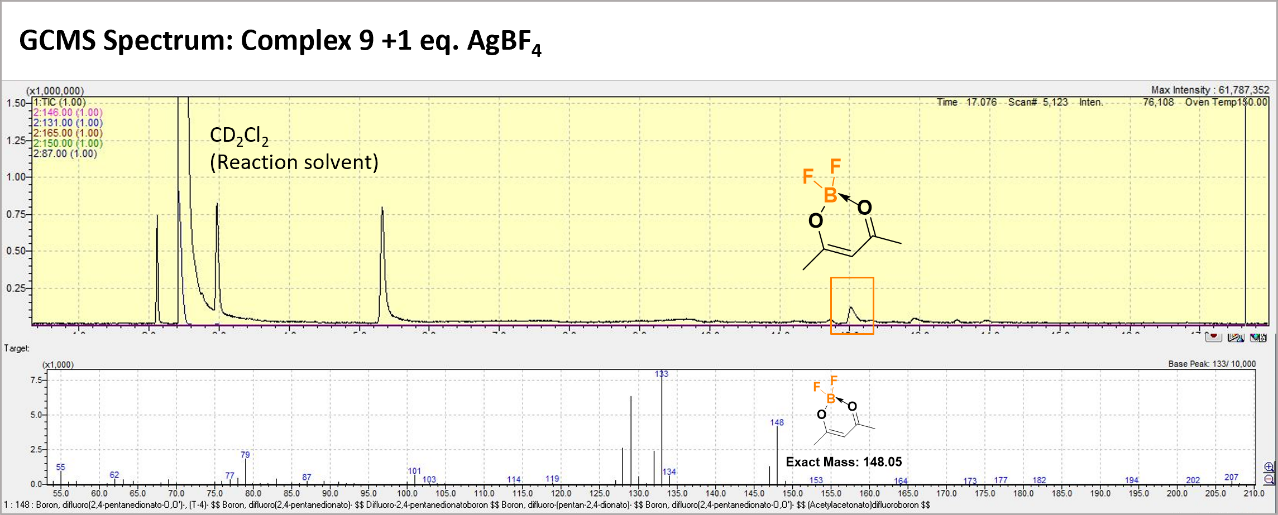
**

**Figure S7**. GCMS spectrum of the reaction mixture of **9** with AgBF_4_.

**
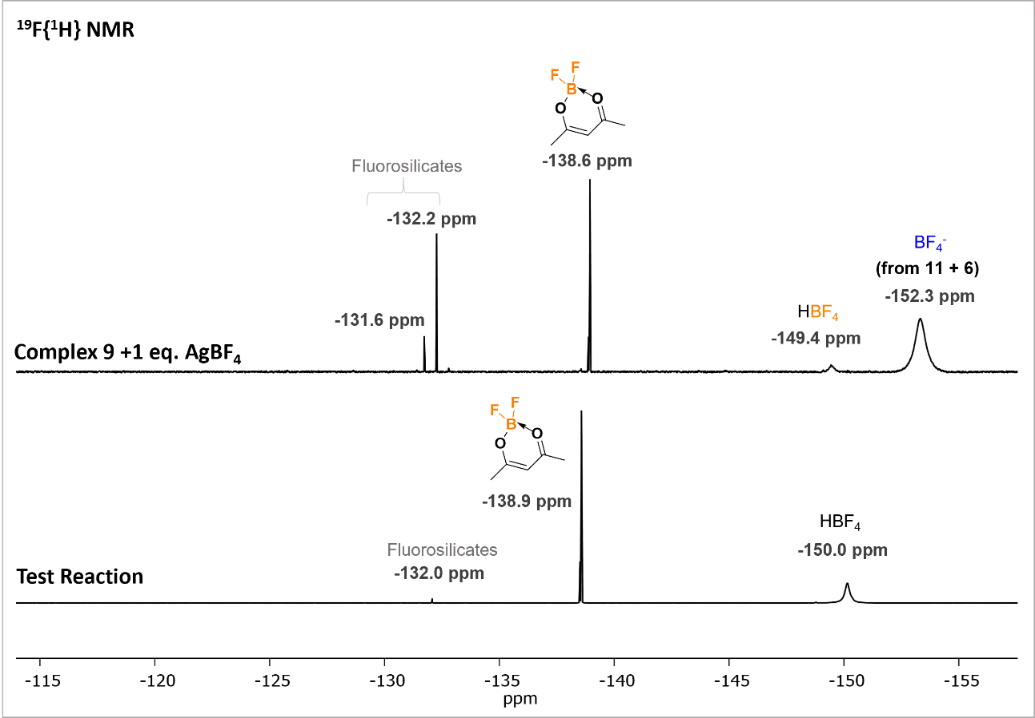
**

**Figure S8**. Comparing ^19^F{^1^H} NMR of the reaction of **9** with AgBF_4_ with test reaction.

*All NMR spectra measured in CD_2_Cl_2_ at 300 MHz.

**
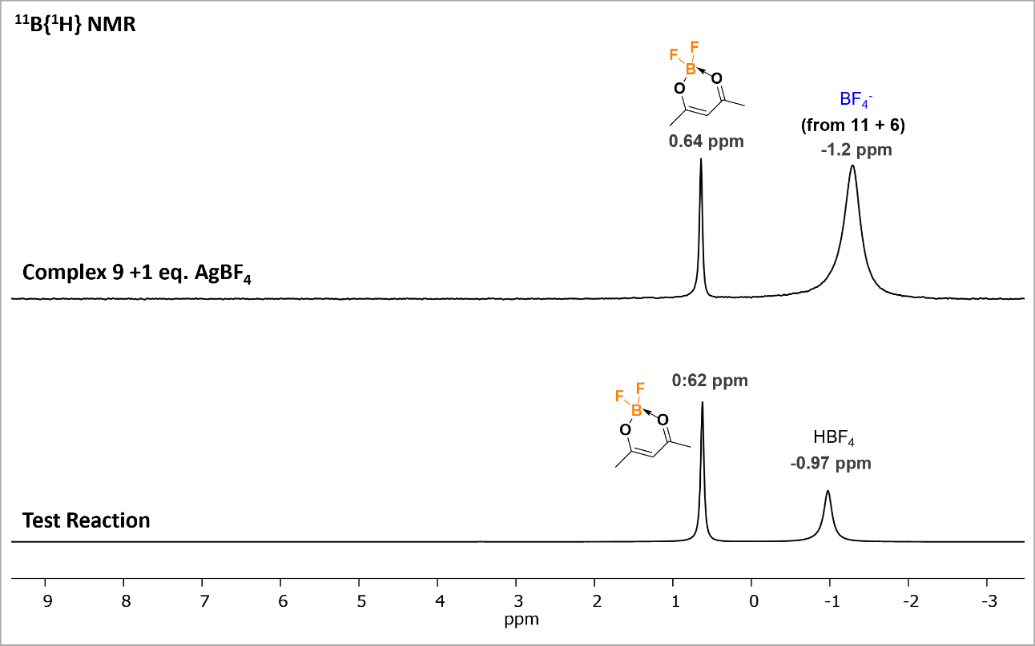
**

**Figure S9**. Comparing ^11^B{^1^H} NMR of the reaction of **9** with AgBF_4_ with test reaction.

*All NMR spectra measured in CD_2_Cl_2_ at 300 MHz.

**3 Low Temperature ^1^H NMR spectra for Complex 13**


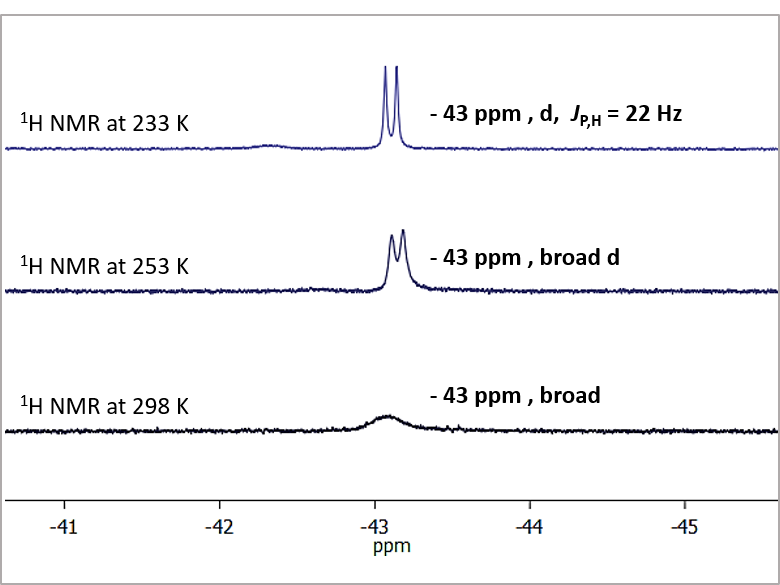


**Figure S10**. Hydride region in ^1^H NMR spectrum at 300 MHz for complex **13**.

*All NMR spectra measured in CD_2_Cl_2_ at 300 MHz

# **4 Procedure for Alkane Transfer Dehydrogenation Reactions**

**For COA:** A stock solution of the catalyst **1** (17 mg, 0.024 mmol) and NaO*t*Bu (14 mg, 0.15 mmol) in 1 mL of C_6_D_6_ was prepared. To a J-Young NMR tube COA (0.3 mL, 2.2 mmol, 3000 eq.), mesitylene (139 *μ*L,1 mmol) as a GC-MS standard, TBE (0.29 mL, 2.2 mmol, 3000 eq.) and 90 *μ*L of stock solution were added. An NMR spectrum was measured and the reaction was heated to 180 °C over 72 hours and then analysed by GC-MS.

The same procedure was repeated for all alkane substrates.

**For unsaturated heterocycles:** In a J-Young NMR tube, were weighed the catalyst **1** (8.3 mg, 12 *μ*mol, 1 eq.) and NaO*t*Bu (6.9 mg, 72 *μ*mol, 6 eq.). The respective substrate (2.4 mmols) and mesitylene (139 *μ*L, 1 mmol) were added and the reaction was heated to 180 °C over 72 hours and analysed by GC-MS.


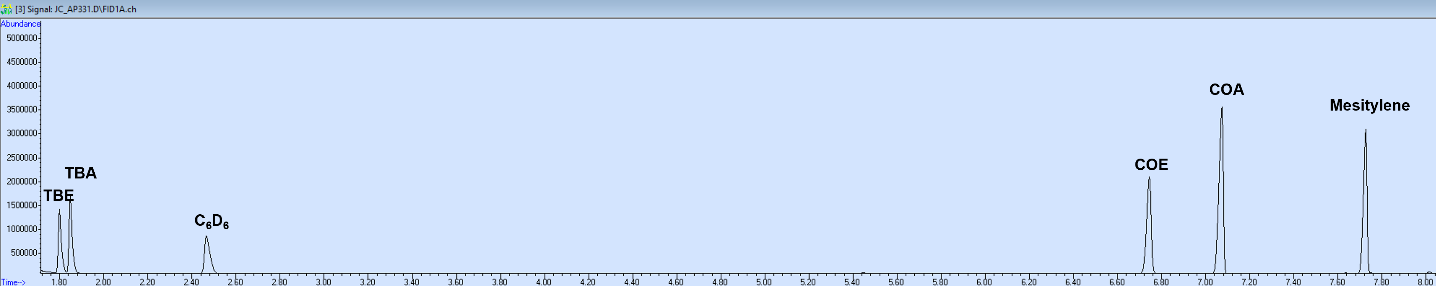


**Figure S11**. Representative GC-MS spectra: Reaction mixture of the transfer dehydrogenation of cyclooctane.

**Calculation of TON**^[1,2]^

TON = [Conversion of Alkane] x [[Alkane]_initial_/ [Catalyst]_initial_]

# **5 Proof of tetrameric structure of [(*^t^*^Bu^POC)Ir(O_2_CCF_3_)(H)]_4_ (2)**

**5.1 IR spectra of 2**


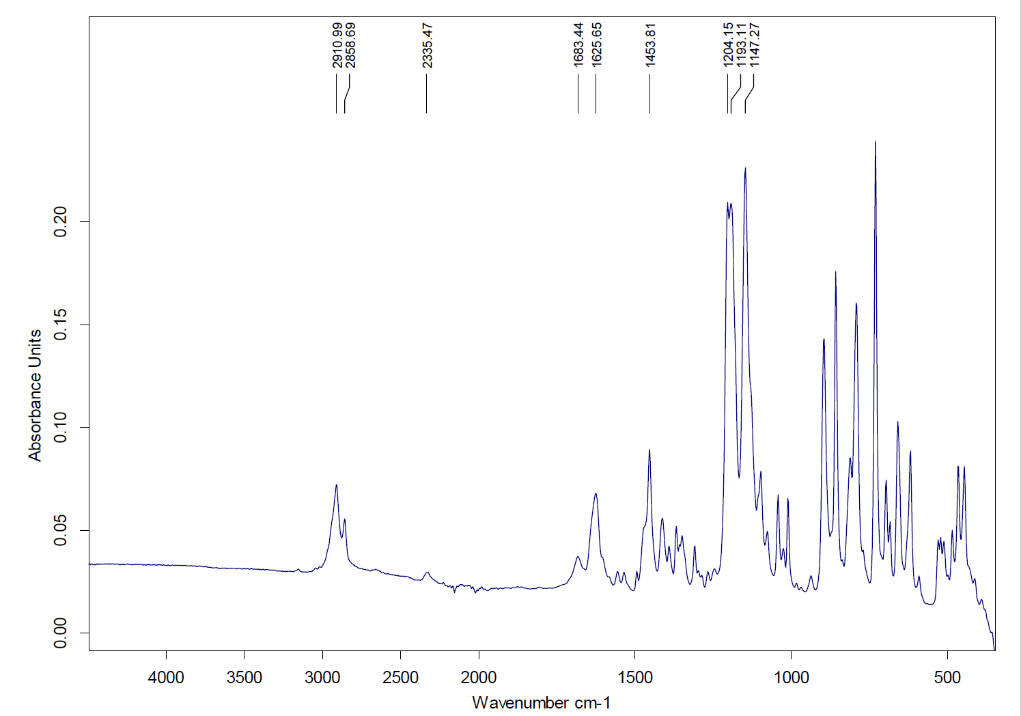


**Figure S12**. IR spectrum of solid **2**.


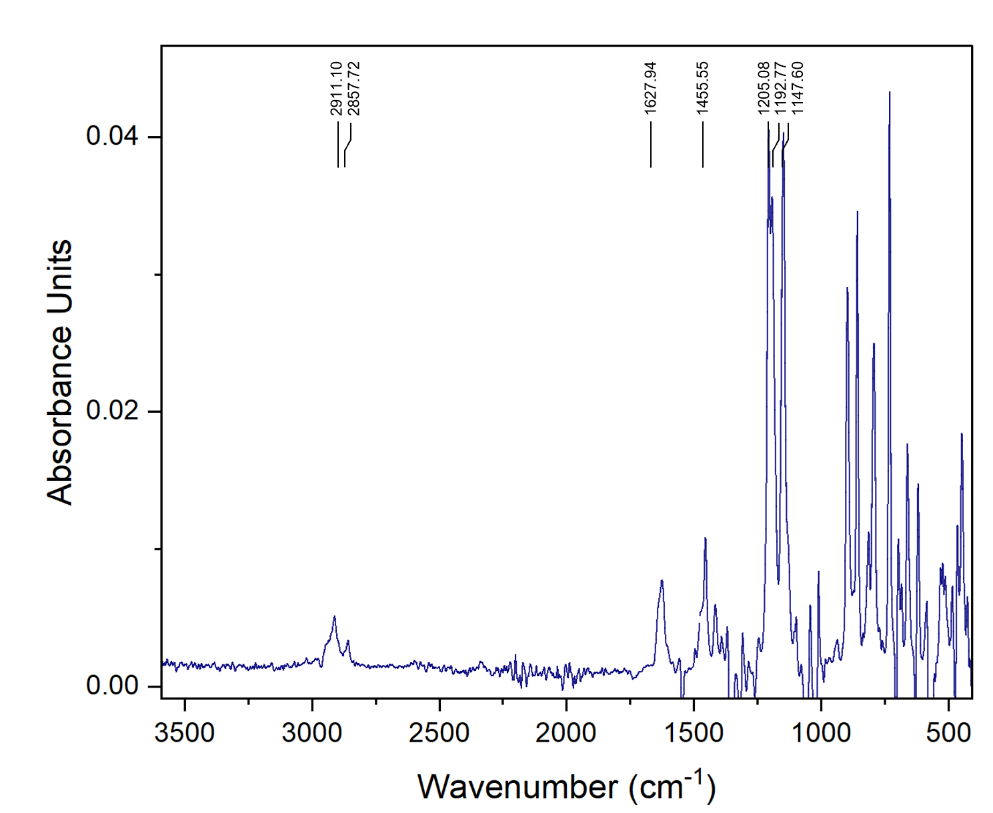


**Figure S13**. IR spectrum of **2** in *o*-dichlorobenzene.

**5.2 ^1^H,^1^H-NOESY spectrum of 2**


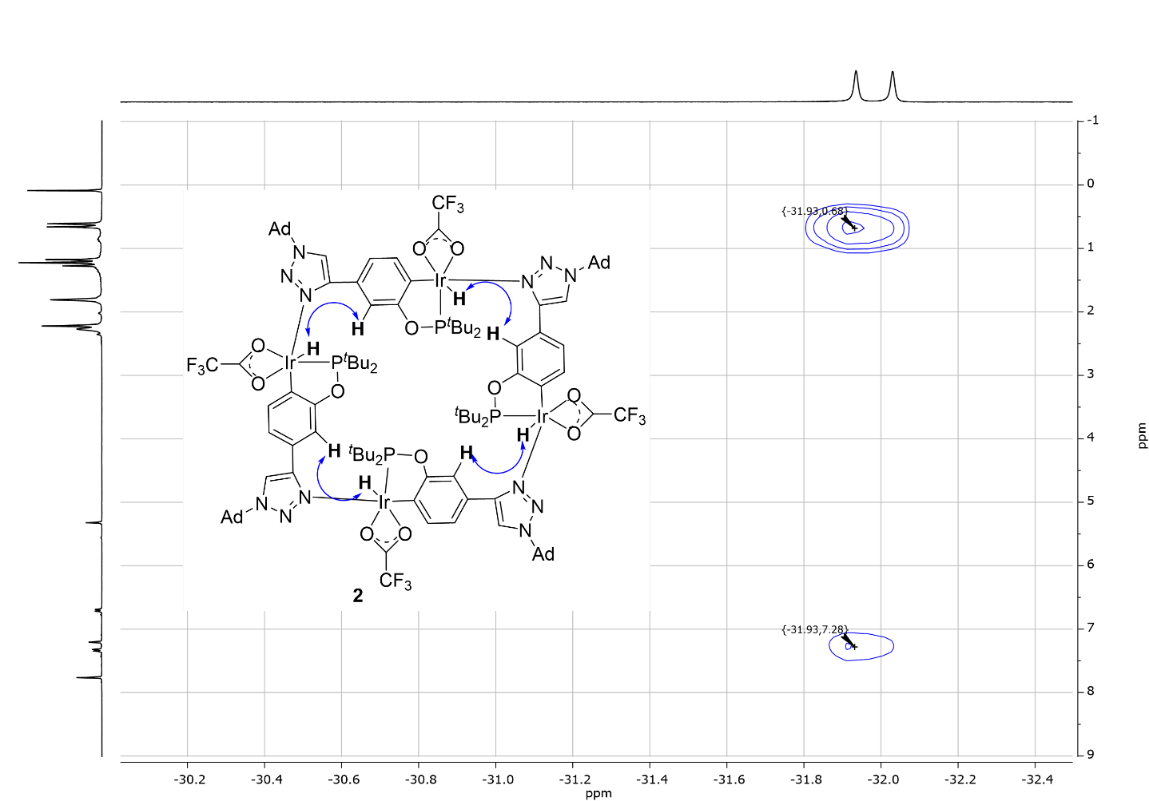


**Figure S14**. ^1^H,^1^H-NOESY NMR spectrum of **2** in CD_2_Cl_2_.

**
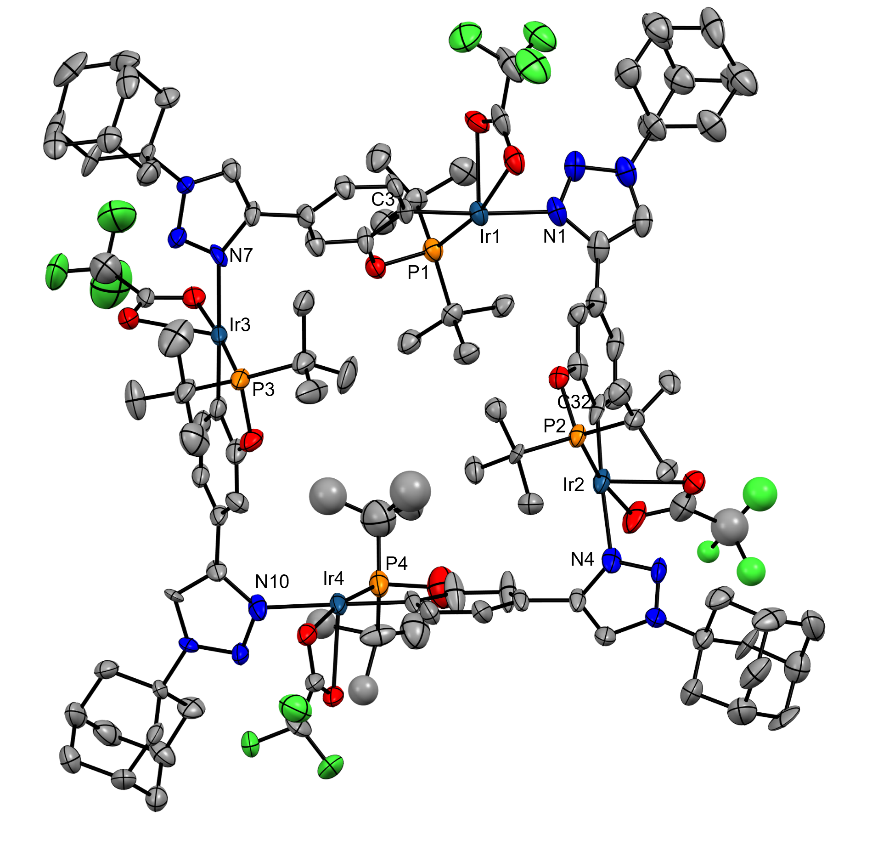
5.3 Molecular Structure of 2**

**Figure S15.** Molecular structure of [(*^t^*^Bu^POC)Ir(O_2_CCF_3_)(H)]_4_· 2CH_2_Cl_2_ (**2**·2CH_2_Cl_2_). Hydrogen atoms and the solvent molecules were omitted for clarity. Thermal ellipsoids are drawn at 50% probability level. Non-hydrogen atoms that have been refined isotropically, due to the rather poor quality of the crystal, are depicted as balls.

# **6 Cyclic Voltammetry**

Cyclic voltammograms of **1** and **3** were recorded in DCM as solvent, with a glassy carbon working electrode and an Ag/Ag^+^ reference electrode. All potentials are referenced vs the Fc^+/o^ couple.

**
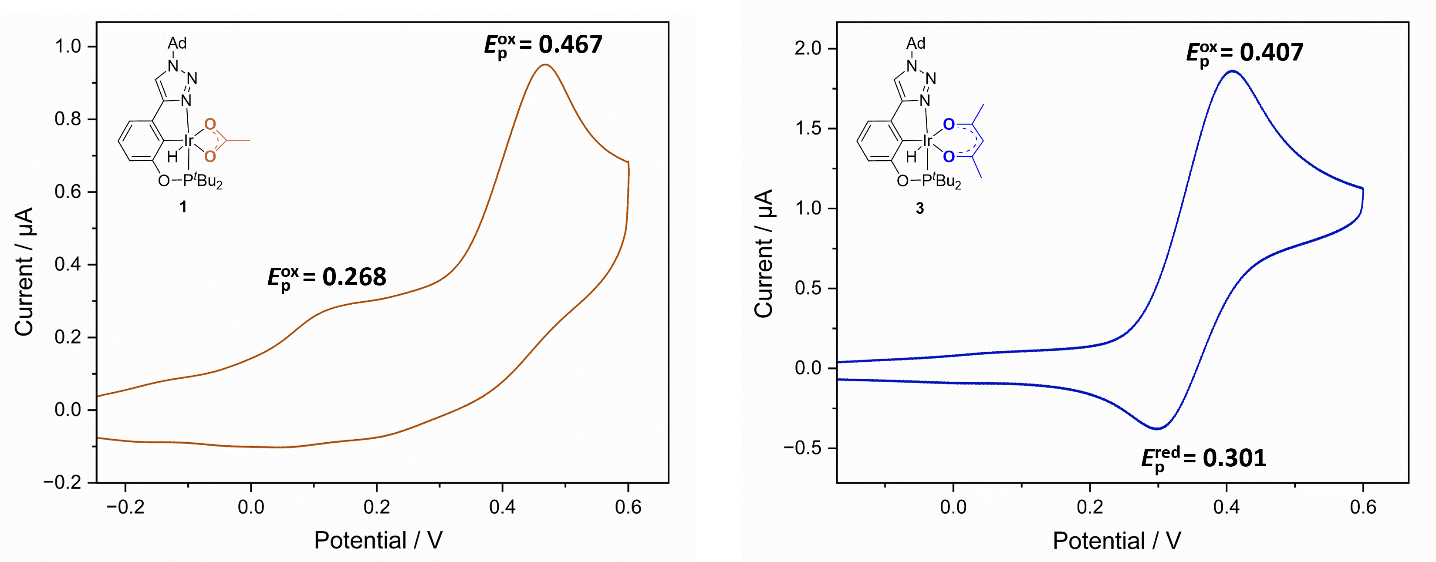
**

**Figure S16**. Cyclic voltammograms of **1** and **3**, 1 mM, 0.1 M [NBu_4_][PF_6_], 25 °C, 100 mV/s.

For **3** additional cyclic voltammograms were recorded with increasing amounts of acetic acid. This led to changes in the current corresponding to the reduction wave, making the wave more reversible as the concentration of acid increased.





**Figure S17**. Cyclic voltammograms of **3** (1 mM) in presence of increasing equivalents of acetic acid in dichloromethane, 0.1 M [NBu_4_][PF_6_], 25 °C, 100 mV/s.

# **7 Reactivity of [(*^t^*^Bu^POCN_Triaz_)Ir(H)(O_2_CCH_3_)]** **(1) with other reagents**

The reactivity of **1** was tested towards the following reagents at room temperature in CD_2_Cl_2_. All reactions were analyzed for up to 20 hours.

| **Reagent** | **Result** | **^31^P{^1^H} NMR** |
| --- | --- | --- |
| 1eq. [NaBAr^F^] | No reaction | 157.7 ppm |
| 1eq. AgBF_4_ | Complex **6** | 86. 7 ppm |
| 1eq. AgPF_6_ | Complex **6** (with PF_6_^-^ counter anion) | 84.5 ppm |
| 1eq. [NO][BF_4_] | Decomposition | No signal |
| 1eq. TlPF_6_ | No reaction* | 157.7 ppm |

*Reaction was attempted in CD_2_Cl_2_ as well as THF-*d8*

# **8 Reactivity of [(*^t^*^Bu^POCN_Triaz_)Ir(H)(acac)]** **(3) with other reagents**

The reactivity of **3** was tested towards the following reagents at room temperature in CD_2_Cl_2_. All reactions were analyzed for up to 20 hours.

| **Reagent** | **Result** | **^31^P{^1^H} NMR** |
| --- | --- | --- |
| CO | No reaction | 156.7 ppm |
| 1 eq. NaO*t*Bu + CO | No reaction^§^ | 156.7 |

^§^Reaction occurs at 60 °C, but not at room temperature.

**9 X-Ray Crystallographic Data**

Structure determination of complex **3**: Colorless plates were obtained by slow evaporation of the *n*-pentane washings of complex **3**. The diffraction data were collected at a Bruker D8 Venture diffractometer at 100 K using Mo-Kα (λ = 0.71073 Å) radiation. Multi-scan absorption corrections implemented SADABS were applied to the data. The structures were solved by intrinsic phasing method (SHELXT 2014/5)^[3]^ and refined y full-matrix least-squares methods on F2 (SHELXL 2016/4 or SHELXL-2018/3).^[4]^

**Table S1. Crystal data and structure refinement for [(POCN)Ir(acac)(H)] (1):**

| Identification code | mo_o24_JC_0m_a |
| --- | --- |
| Empirical formula | C_31_H_45_IrNO_3_P |
| Formula weight | 729.86 |
| Temperature | 150(2) K |
| Wavelength | 0.71073 Å |
| Crystal system | Monoclinic |
| Space group | *C*2*/c* |
| Unit cell dimensions | a = 34.248(2) Å |
|  | b = 11.5964(8) Å |
|  | c = 17.2271(9) Å |
| Volume | 6155.5(7) Å^3^ |
| Z | 8 |
| Density (calculated) | 1.575 Mg/m^3^ |
| Absorption coefficient | 4.425 mm^-1^ |
| F(000) | 2936 |
| Crystal size | 0.420 x 0.382 x 0.022 mm^3^ |
| Theta range for data collection | 2.37 to 26.06 ° |
| Index ranges | -41<=h<=41, -14<=k<=14, -21<=l<=21 |
| Reflections collected | 179291 |
| Independent reflections | 5797 [R(int) = 0.1247] |
| Completeness to theta = 25.242° | 99.1 % |
| Absorption correction | Multi-scan |
| Max. and min. transmission | 0.4908 and 0.4028 |
| Refinement method | Full-matrix least-squares on F^2^ |
| Data / restraints / parameters | 5797 / 6 / 367 |
| Goodness-of-fit on F^2^ | 1.055 |
| Final R indices [I>2sigma(I)] | R1 = 0.0325, wR2 = 0.0860 |
| R indices (all data) | R1 = 0.0412, wR2 = 0.0810 |
| Largest diff. peak and hole | 2.446 and -0.760 e.Å^-3^ |
| CCDC | 2468854 |

Structure determination of complex **2**: Yellow plates were obtained by layering a saturated solution of **2** in DCM with *n*-pentane.

**Table S2. Crystal data and structure refinement for [(*^t^*^Bu^POC)Ir(O_2_CCF_3_)(H)]_4_ (2):**

| Identification code | mo_o25_JCdh425_a |
| --- | --- |
| Empirical formula | C_104_H_138_F_12_Ir_4_N_12_O_12_P_6_ |
| Formula weight | 3148.98 |
| Temperature | 100(2) K |
| Wavelength | 0.71073 Å |
| Crystal system | Triclinic |
| Space group | *P*$\bar{1}$ |
| Unit cell dimensions | a = 17.222(2) Å |
|  | b = 19.781(3) Å |
|  | c = 23.952(3) Å |
| Volume | 7384.8(16) Å^3^ |
| Z | 2 |
| Density (calculated) | 1.416 Mg/m^3^ |
| Absorption coefficient | 3.775 mm^-1^ |
| F(000) | 3128 |
| Crystal size | 0.391 x 0.145 x 0.019 mm^3^ |
| Theta range for data collection | 2.2100 to 25.3856° |
| Index ranges | -20<=h<=20, -23<=k<=34, -28<=l<=29 |
| Reflections collected | 356221 |
| Independent reflections | 27459 [R(int) = 0.1527] |
| Completeness to theta = 25.242° | 99.9 % |
| Absorption correction | Multi-scan |
| Max. and min. transmission | 0.932 and 0.320 |
| Refinement method | Full-matrix least-squares on F^2^ |
| Data / restraints / parameters | 27459 / 85 / 1447 |
| Goodness-of-fit on F^2^ | 1.116 |
| Final R indices [I>2sigma(I)] | R1 = 0.1076, wR2 = 0.3190 |
| R indices (all data) | R1 = 0.1473, wR2 = 0.2868 |
| Largest diff. peak and hole | 5.438 and -7.377 e.Å^-3^ |
| CCDC | 2531616 |

**10 NMR Spectra**

**Figure S18**. ^1^H NMR spectrum of 3-(1-adamantan-1-yl)-1*H*-1,2,3-triazol-4-yl)phenol.

**Figure S19**. ^13^C{^1^H} NMR spectrum of 3-(1-adamantan-1-yl)-1*H*-1,2,3-triazol-4-yl)phenol..

**Figure S20**. ^1^H NMR spectrum of *^t^*^Bu^POCN_Triaz_ ligand.

**Figure S21.** ^13^C{^1^H} NMR spectrum of *^t^*^Bu^POCN_Triaz_ ligand.

**Figure S22.** ^31^P{^1^H} NMR spectrum of *^t^*^Bu^POCN_Triaz_ ligand.

**Figure S23.** ^1^H NMR spectrum of [(*^t^*^Bu^POCN_Triaz_)Ir((O_2_CCH_3_)(H)] (**1**).

**Figure S24.** ^13^C{^1^H} NMR spectrum of [(*^t^*^Bu^POCN_Triaz_)Ir((O_2_CCH_3_)(H)] (**1**).

**Figure S25.** ^31^P{^1^H} NMR spectrum of [(*^t^*^Bu^POCN_Triaz_)Ir((O_2_CCH_3_)(H)] (**1**).

**Figure S26.** ^1^H NMR spectrum of [(*^t^*^Bu^POC)Ir(O_2_CCF_3_)(H)]_4_ (**2**).

**Figure S27.** ^13^C{^1^H} NMR spectrum of [(*^t^*^Bu^POC)Ir(O_2_CCF_3_)(H)]_4_ (**2**).

**Figure S28.** ^31^P{^1^H} NMR spectrum of [(*^t^*^Bu^POC)Ir(O_2_CCF_3_)(H)]_4_ (**2**).

**Figure S29.** ^19^F{^1^H} NMR spectrum of [(*^t^*^Bu^POC)Ir(O_2_CCF_3_)(H)]_4_ (**2**).

**Figure S30.** ^1^H NMR spectrum of [(*^t^*^Bu^POCN_Triaz_)Ir(acac)(H)] (**3**).

**Figure S31.** ^13^C{^1^H} NMR spectrum of [(*^t^*^Bu^POCN_Triaz_)Ir(acac)(H)] (**3**).

**Figure S32.** ^31^P{^1^H} NMR spectrum of [(*^t^*^Bu^POCN_Triaz_)Ir(acac)(H)] (**3**).

**Figure S33.** ^1^H NMR spectrum of [(*^t^*^Bu^POCN_Triaz_)Ir(CO)] (**4**).

**Figure S34.** ^13^C{^1^H} NMR spectrum of [(*^t^*^Bu^POCN_Triaz_)Ir(CO)] (**4**).

**Figure S35.** ^31^P{^1^H} NMR spectrum of [(*^t^*^Bu^POCN_Triaz_)Ir(CO)] (**4**).

**Figure S36.** ^1^H NMR spectrum of [(*^t^*^Bu^POC)Ir(CO)_2_] (**5**).

**Figure S37.** ^13^C{^1^H} NMR spectrum of [(*^t^*^Bu^POC)Ir(CO)_2_] (**5**).

**Figure S38.** ^31^P{^1^H} NMR spectrum of [(*^t^*^Bu^POC)Ir(CO)_2_] (**5**).

**Figure S39.** ^1^H NMR spectrum of Complex **6**.

**Figure S40.**^1^H,^13^C-HSQC spectrum of Complex **6**.

**Figure S41.**^1^H,^13^C-HMBC spectrum of Complex **6**.

**Figure S42.** ^31^P{^1^H} NMR spectrum of Complex **6.**

**Figure S43.** ^19^F{^1^H} NMR spectrum of Complex **6**.

**Figure S44.** ^11^B{^1^H} NMR spectrum of Complex **6**.

**Figure S45.** ^1^H NMR spectrum of Complex **7**.

**Figure S46.** ^13^C{^1^H} NMR spectrum of Complex **7**.

**Figure S47.** ^31^P{^1^H} NMR spectrum of Complex **7**.

**Figure S48.** ^19^F{^1^H} NMR spectrum of Complex **7**.

**Figure S49.** ^11^B {^1^H} NMR spectrum of Complex **7**.

**Figure S50.** ^1^H NMR spectrum of Complex **8**.

**Figure S51.** ^13^C{^1^H} NMR spectrum of Complex **8**.

**Figure S52.** ^13^C,^1^H HSQC spectrum of Complex **8**.

**Figure S53.** ^31^P{^1^H} NMR spectrum of Complex **8**.

**Figure S54.** ^19^F{^1^H} NMR spectrum of Complex **8.**

**Figure S55.** ^11^B {^1^H} NMR spectrum of Complex **8.**

**Figure S56.** ^1^H NMR spectrum of [(*^t^*^Bu^POCN_Triaz_)Ir(H)(acac)•AgBF_4_] (**9**).

**Figure S57.** ^1^H,^13^C - HSQC NMR spectrum of [(*^t^*^Bu^POCN_Triaz_)Ir(H)(acac)•AgBF_4_] (**9**).

**Figure S58.** ^31^P{^1^H} NMR spectrum of [(*^t^*^Bu^POCN_Triaz_)Ir(H)(acac)•AgBF_4_] (**9**).

**Figure S59.** ^19^F{^1^H} NMR spectrum of [(*^t^*^Bu^POCN_Triaz_)Ir(H)(acac)•AgBF_4_] (**9**).

**Figure S60.** ^11^B{^1^H} NMR spectrum of [(*^t^*^Bu^POCN_Triaz_)Ir(H)(acac)•AgBF_4_] (**9**).

**Figure S61.** ^1^H NMR spectrum of [(*^t^*^Bu^POCN_Triaz_)Ir(H)(CO)] [BF_4_] (**10**).

**Figure S62.** ^13^C{^1^H} NMR spectrum of [(*^t^*^Bu^POCN_Triaz_)Ir(H)(CO)] [BF_4_] (**10**).

**Figure S63.** ^31^P{^1^H} NMR spectrum of [(*^t^*^Bu^POCN_Triaz_)Ir(H)(CO)] [BF_4_] (**10**).

**Figure S64.** ^19^F{^1^H} NMR spectrum of [(*^t^*^Bu^POCN_Triaz_)Ir(H)(CO)] [BF_4_] (**10**).

**Figure S65.** ^11^B{^1^H} NMR spectrum of [(*^t^*^Bu^POCN_Triaz_)Ir(H)(CO)] [BF_4_] (**10**).

**Figure S66.** ^1^H NMR spectrum of [(*^t^*^Bu^POCN_Triaz_)Ir(acac)] [BF_4_] (**11**).

**Figure S67.** ^13^C{^1^H} NMR spectrum of [(*^t^*^Bu^POCN_Triaz_)Ir(acac)] [BF_4_] (**11**).

**Figure S68.** ^31^P{^1^H} NMR spectrum of [(*^t^*^Bu^POCN_Triaz_)Ir(acac)] [BF_4_] (**11**).

**Figure S69.** ^19^F{^1^H} NMR spectrum of [(*^t^*^Bu^POCN_Triaz_)Ir(acac)] [BF_4_] (**11**).

**Figure S70.** ^1^H NMR spectrum of [(*^t^*^Bu^POCN_Triaz_)Ir(acac)(CO)] [BF_4_] (**12**).

**Figure S71.** ^13^C{^1^H} NMR spectrum of [(*^t^*^Bu^POCN_Triaz_)Ir(acac)(CO)] [BF_4_] (**12**).

**Figure S72.** ^31^P{^1^H} NMR spectrum of [(*^t^*^Bu^POCN_Triaz_)Ir(acac)(CO)] [BF_4_] (**12**).

**Figure S73.** ^19^F{^1^H} NMR spectrum of [(*^t^*^Bu^POCN_Triaz_)Ir(acac)(CO)] [BF_4_] (**12**).

**Figure S74.** ^11^B{^1^H} NMR spectrum of [(*^t^*^Bu^POCN_Triaz_)Ir(acac)(CO)] [BF_4_] (**12**).

**Figure S75.** ^1^H NMR spectrum of [(*^t^*^Bu^POCN_Triaz_)Ir(H)(OAc)•B(C_6_F_5_)_3_] (**13**).

**Figure S76.** ^1^H,^13^C-HSQC NMR spectrum of [(*^t^*^Bu^POCN_Triaz_)Ir(H)(OAc)•B(C_6_F_5_)_3_] (**13**).

**Figure S77.** ^31^P{^1^H} NMR spectrum of [(*^t^*^Bu^POCN_Triaz_)Ir(H)(OAc)•B(C_6_F_5_)_3_] (**13**).

**Figure S78.** ^19^F{^1^H} NMR spectrum of [(*^t^*^Bu^POCN_Triaz_)Ir(H)(OAc)•B(C_6_F_5_)_3_] (**13**).

**Figure S79.** ^11^B{^1^H} NMR spectrum of [(*^t^*^Bu^POCN_Triaz_)Ir(H)(OAc)•B(C_6_F_5_)_3_] (**13**).

**Figure S80.** ^1^H NMR spectrum of [(*^t^*^Bu^POCN_Triaz_)Ir(H)(2,2´-bipy)] [B(OAc)(C_6_F_5_)_3_] (**14**).

**Figure S81.** ^13^C{^1^H} NMR spectrum of [(*^t^*^Bu^POCN_Triaz_)Ir(H)(2,2´-bipy)] [B(OAc)(C_6_F_5_)_3_] (**14**).

**Figure S82.** ^31^P{^1^H} NMR spectrum of [(*^t^*^Bu^POCN_Triaz_)Ir(H)(2,2´-bipy)] [B(OAc)(C_6_F_5_)_3_] (**14**).

**Figure S83.** ^19^F{^1^H} NMR spectrum of [(*^t^*^Bu^POCN_Triaz_)Ir(H)(2,2´-bipy)] [B(OAc)(C_6_F_5_)_3_] (**14**).

**Figure S84.** ^11^B {^1^H} NMR spectrum of [(*^t^*^Bu^POCN_Triaz_)Ir(H)(2,2´-bipy)] [B(OAc)(C_6_F_5_)_3_] (**14**).

**11 References**

[1] X. Zhou, S. Malakar, T. Dugan, K. Wang, A. Sattler, D. O. Marler, T. J. Emge, K. Krogh-Jespersen, A. S. Goldman, *ACS Catal.* **2021**, *11*, 14194–14209.

[2] J. Cardozo, O. He, W. Ma, K. Ray, T. Braun, *Zeitschrift anorg allge chemie* **2024**, *650*, DOI 10.1002/zaac.202400028.

[3] G. M. Sheldrick, *Acta Crystallogr A Found Adv* **2015**, *71*, 3–8.

[4] G. M. Sheldrick, *Acta Crystallogr C Struct Chem* **2015**, *71*, 3–8.
